# TSPO expression in a Zika virus murine infection model as an imaging target for acute infection-induced neuroinflammation

**DOI:** 10.1007/s00259-022-06019-w

**Published:** 2022-11-09

**Authors:** Carla Bianca Luena Victorio, Rasha Msallam, Wisna Novera, Joanne Ong, Tham Jing Yang, Arun Ganasarajah, Jenny Low, Satoru Watanabe, Ann-Marie Chacko

**Affiliations:** 1 Laboratory for Translational and Molecular Imaging, Cancer and Stem Cell Biology Programme, Duke-NUS Medical School, 8 College Road, Singapore, 169857 Singapore; 2 Department of Infectious Diseases, Singapore General Hospital, 20 College Road, Singapore, 169856, Singapore; 3 Programme in Emerging Infectious Disease, Duke-NUS Medical School, 8 College Road, Singapore, 169857, Singapore

**Keywords:** Zika, Neuroinflammation, Translocator protein, TSPO, PET

## Abstract

**Introduction:**

Zika virus (ZIKV) is a neurotropic human pathogen that causes neuroinflammation, whose hallmark is elevated translocator protein (TSPO) expression in the brain. This study investigates ZIKV-associated changes in adult brain TSPO expression, evaluates the effectiveness of TSPO radioligands in detecting TSPO expression, and identifies cells that drive brain TSPO expression in a mouse infection model.

**Methods:**

The interferon-deficient AG129 mouse infected with ZIKV was used as neuroinflammation model. TSPO expression was evaluated by tissue immunostaining. TSPO radioligands, [^3^H]PK11195 and [^18^F]FEPPA, were used for in vitro and ex vivo detection of TSPO in infected brains. [^18^F]FEPPA-PET was used for in vivo detection of TSPO expression. Cell subsets that contribute to TSPO expression were identified by flow cytometry.

**Results:**

Brain TSPO expression increased with ZIKV disease severity. This increase was contributed by TSPO-positive microglia and infiltrating monocytes; and by influx of TSPO-expressing immune cells into the brain. [^3^H]PK11195 and [^18^F]FEPPA distinguish ZIKV-infected brains from normal controls in vitro and ex vivo. [^18^F]FEPPA brain uptake by PET imaging correlated with disease severity and neuroinflammation. However, TSPO expression by immune cells contributed to significant blood pool [^18^F]FEPPA activity which could confound [^18^F]FEPPA-PET imaging results.

**Conclusions:**

TSPO is a biologically relevant imaging target for ZIKV neuroinflammation. Brain [^18^F]FEPPA uptake can be a surrogate marker for ZIKV disease and may be a potential PET imaging marker for ZIKV-induced neuroinflammation. Future TSPO-PET/SPECT studies on viral neuroinflammation and related encephalitis should assess the contribution of immune cells on TSPO expression and employ appropriate image correction methods to subtract blood pool activity.

**Supplementary Information:**

The online version contains supplementary material available at 10.1007/s00259-022-06019-w.

## Introduction

Zika virus (ZIKV) is a neurotropic virus, and human infection often results in neuroinflammation with devastating neurologic consequences. It is a member of the *Flaviviridae* family of positive-sense, single-stranded RNA genome viruses that was first isolated in the Ugandan Zika Forest [1]. While reported as neurotropic in experimental animal infection models and brain-derived in vitro culture systems (reviewed in [2]), ZIKV-associated neurological manifestations in humans were not reported until the first outbreak on Yap Island in 2007 [3]. Subsequently, a surge in fetal microcephaly and adult Guillain-Barré syndrome cases were found associated with ZIKV outbreaks in Brazil [4] and Latin America. Other less common neurological complications associated with ZIKV infections include acute transverse myelitis [5–7], encephalomyelitis [8, 9], acute disseminated encephalomyelitis [10], and severe meningoencephalitis [11].

Neuroinflammation is a complex response orchestrated by reactive cells in the central nervous system (CNS) to altered homeostasis, which results from insults either internal or external to the CNS. These reactive cells are primarily comprised of brain-resident glia, astrocytes, oligodendrocytes, and infiltrating immune cells, which are all invariably activated in CNS diseases. One of the hallmarks of neuroinflammation is the increased CNS expression of translocator protein (TSPO)—a mitochondrial protein basally expressed by microglia, astrocytes, neural stem cells, pericytes, and endothelial cells in normal healthy mouse brains [12]. Elevated TSPO expression in rodent models has been successfully imaged non-invasively in various CNS disturbances, including Alzheimer’s disease, stroke, and traumatic brain injury using TSPO-binding radioligands [13–15] (recently reviewed in [16]). TSPO radioligands have also been used in PET imaging of virus infection-induced neuroinflammation in animal models, including ZIKV [17–20]. Kuszpit et. al. showed increased [^18^F]DPA-714 PET signals in ZIKV mouse brains but did not correlate PET signal enhancement with brain TSPO expression [20]. Moreover, recent studies have argued against the traditional view of pathogenic microglia as the sole source of TSPO expression in the inflamed CNS and uncovered astrocytes, neurons, and endothelia as other contributing TSPO-expressing cells (reviewed in [21, 22]). Whether immune cells other than microglia in the CNS also contribute to elevated TSPO expression in the brains of ZIKV-diseased mice—and consequently to increased binding of TSPO radioligands—is currently not known.

The study described herein was designed to evaluate changes in TSPO expression in ZIKV disease, to correlate TSPO radioligand binding with ZIKV disease, and to further identify which cells in the CNS contribute to elevated TSPO expression. An AG129 mouse strain deficient in interferon (IFN) receptor expression was employed in an acute adult ZIKV infection model distinct from the one studied by Kuszpit et. al. [20]. The AG129 model is widely used for studying ZIKV disease pathology and pathogenesis [23–27] since functional IFN signaling blocks ZIKV infection, and wild-type mice do not exhibit overt ZIKV disease (reviewed in [28–30]). We chose two different time points post-infection to demonstrate disease-dependent increase in binding of TSPO radioligands [^3^H]PK11195 and [^18^F]FEPPA using tissue autoradiography and ex vivo tissue biodistribution studies. Moreover, quantitative flow cytometry was used to demonstrate that TSPO expression in the ZIKV brain is not restricted to CNS resident microglia and astrocytes, but also includes immune cells mobilized from the periphery to increase total TSPO expression—and thereby increasing binding by TSPO radioligands. Lastly, we report on the possibility of using [^18^F]FEPPA-PET for detection of ZIKV-driven TSPO expression in the brain, while highlighting the importance of delineating confounding factors in PET imaging interpretation, such as on-target TSPO radioligand binding to immune cells within the vascular circulation.

## Materials and methods

### Radiotracers

[^3^H]PK11195 (NET885250UC, 9.25 MBq, 2912 GBq/mmol) was purchased from Perkin-Elmer, Singapore. [^18^F]FEPPA (4.24 GBq, 533 GBq/μmol) was synthesized at > 99% radiochemical purity by National University of Singapore Clinical Imaging Research Center (NUS-CIRC).

### Animal infection

All animal experiments were conducted with approval from the Institutional Animal Care and Use Committee (IACUC) of Duke-NUS Medical School and Singhealth (IACUC approval no. 2020/SHS/1607) and conformed to the National Institutes of Health (NIH) guidelines and public law. The animals were housed in individually ventilated cages and provided with food pellets and water ad libitum.

The ZIKV virus strain Paraiba01/2015 (Genbank Accession No. KX280026.1) was isolated from an anonymous patient in the state of Paraiba, Brazil and was a kind gift from Dr. Pedro Vasconcelos (Instituto Evandro Chagas, Brazil). The AG129 mice (adult male) deficient in expression of IFN-α/β and γ receptors were obtained from an in-house breeding colony.

The ZIKV infection model was established as described previously [27, 31]. Briefly, mice were infected with ZIKV by intraperitoneal (i.p.) injection of 10^5^ plaque-forming units (PFU) in 100 μL. Mock-infected controls were similarly inoculated with an equal volume of sterile phosphate buffered saline (PBS, pH 7.4). Both groups of animals were observed daily for signs of disease and underwent general health and neurological examinations. ZIKV-infected mice were euthanized when moribund, based on these criteria: (1) loss of > 20% original body weight, (2) persistent limb paralysis of > 48 h, and (3) immobility or severe lethargy. Animals were euthanized by 10% CO_2_ inhalation for 5 min.

### In vitro assays of neuroinflammation

Whole brains were harvested as described previously [32]. For immunohistopathological evaluation, brains were fixed in 10% neutral buffered formalin (Sigma-Aldrich, USA) for 72 h and processed for embedding in OCT. Tissue cryosections (10 μm thick) were incubated overnight with either rabbit antibody against translocator protein (TSPO) (Invitrogen MA5-31,966) or rabbit antibody against glial fibrillary acidic protein (GFAP) (Dako Agilent GA524) followed by secondary antibody incubation. Stained tissue sections were imaged using Leica Cell DIVE (Leica Microsystems, Singapore).

Brains used for in vitro TSPO-binding with [^3^H]PK11195 were freshly embedded in OCT without tissue fixation and rapidly frozen. Cryosections (20 μm thick) were dried in 37 °C for 1 h prior to rehydration in high salt buffer (0.17 M Tris–Cl, pH 7.4) for 30 min. Tissues were incubated with radiotracer (0.01 mM; 0.087 MBq) in room temperature for 1 h and subsequently washed 3 × with high salt buffer and twice with ice-cold Milli-Q water. Slides were air-dried overnight. Digital autoradiography (DAR) images were acquired for 48 h using BeaVacq (Ai4R, France) and subsequently analyzed using the BeaQuant (Ai4R, France).

Frozen brains were homogenized to extract either total protein or total RNA. Total protein was subjected to ELISA for detection of IL-6 and TNF-α expression using the Ready-Set-GO! Kit following the manufacturer’s protocol (eBioscience, USA). Cytokine expression was reported as pg protein per mg (pg/mg) tissue. Viral RNA was isolated using RNEasy RNA extraction kit (Qiagen, Germany) according to manufacturer’s instructions. Real-time RT-PCR was carried out with primers targeting the E gene of ZIKV genome (ZK_F: 5′- CCGCTGCCCAACACAAG– 3′; ZK_R: 5′- CCACTAACGTTCTTTTGCAGACAT- 3′) as previously described [33]. In vitro transcribed RNA product of ZIKV-H/PF/2013 (accession number: KJ776791.2) E gene was used to generate the standard curve for quantification of viral genome copy.

### Ex vivo biodistribution studies

[^18^F]FEPPA (2 MBq) was injected into the retroorbital space, and mice were sacrificed following 2 h uptake. Tissues were harvested and brains were micro-dissected as described previously [34]. Tissues were immediately loaded into a gamma counter (2470 Wizard2, PerkinElmer, USA). Tracer distribution was reported as % injected dose per gram tissue (% ID/g).

### TSPO imaging by Positron Emission Tomography (PET) and DAR

PET/CT imaging in mice was performed as described previously [27, 35]. Briefly, mice were sedated with 2% isoflurane, and intravenously (i.v.) injected with [^18^F]FEPPA (30 MBq). Following a 2-h tracer uptake phase, mice underwent head-focused PET scan for 10 min (VECTOR^4^CT, MILabs, Netherlands) using the M7 UHR 0.7-mm-pinhole collimator, immediately followed by total-body CT scan for 5 min (tube current, 0.24 mA; tube voltage, 50 kV). The resulting images were reconstructed at 0.8-mm voxel resolution using a SROSEM algorithm with 8 subsets, 14 iterations, and 1.2-μm Gaussian filter. PET and CT images were automatically co-registered, and attenuation correction of the PET image was done using the CT image.

Image analysis was performed using PMOD v3.7 (PMOD Technologies, Switzerland). Volumes of interest (VOIs) were drawn around the brain guided by the skull in CT images. Brain segmentation analysis was performed by drawing VOIs around the cerebral cortex (CTX), diencephalon (Th + Hy), and combined hindbrain (CBX & MY) in the CT images. VOIs were transferred to the co-registered PET images, and tracer uptake was reported as % ID (injected dose).

Following PET imaging, animals were euthanized and brain tissues collected for ex vivo DAR imaging. Cryosections (20 μm) were air-dried for 30 min prior to DAR imaging for 24 h using BeaVacq (Ai4R, France). Images were analyzed using the BeaQuant software (Ai4R, France).

### Flow cytometry

Tissue immune profiling was conducted as previously described [27, 36]. Briefly, brains were harvested, mechanically disaggregated, and incubated in digestion buffer containing DNaseI and collagenase. Following homogenization, single cells were collected by passing the suspension through a nylon mesh strainer (70 μm). Fat was subsequently removed by centrifugation in Percoll. Cells were labelled with fluorescent antibody cocktails, and immune cell subsets were identified by flow cytometry (Fortessa, BD Biosciences, USA) and analyzed using FlowJo V10.8.0 (BD Biosciences, USA). Detected events were normalized to absolute cell number using CountBright Absolute Counting Beads (ThermoFisher, USA) [37]. The following markers were used to identify the immune landscape: total immune cells (CD45^+^); granulocytes (Ly6G^+^Ly6C^+^CD11b^+^); microglia (CD11b^+^F4/80^+^Ly6c^−^Ly6G^−^CD3^−^); monocyte-derived macrophages or mo-MACs (CD11b^+^F4/80^+^Ly6c^+^Ly6G^−^CD3^−^); monocytes (CD11b^+^CD115^+^); dendritic cells (CD11c^+^MHCII^+^); B cells (CD3^−^CD19^+^B220^+^MHCII^+^); total T cells (CD19^−^CD49b^−^B220^−^LY6G^−^CD3^+^); cytotoxic T cells (CD19^−^CD49b^−^B220^−^LY6G^−^CD3^+^CD8^+^), and helper T cells (CD19^−^CD49b^−^B220^−^LY6G^−^CD3^+^CD4^+^). TSPO expression was reported as mean fluorescence intensity (MFI) from samples tagged with anti-mouse TSPO-AlexaFluor 488 (ab199779; Abcam, USA).

### Statistical analysis

Statistical analyses and graphing were performed with Prism v8.0 (GraphPad Software, USA). Means from two groups were compared by Mann–Whitney test, while means from multiple groups were compared by Kruskal–Wallis test with Dunn’s post-hoc correction. Linear regression analyses on scatter plots were performed using Spearman’s coefficient (*ρ*) to assess the correlation between tissue viral load and cytokine expression. Results were considered statistically significant at *p* < 0.05.

## Results

### Neuroinflammation and increased TSPO expression associated with ZIKV neurological infection

**Fig. 1:**
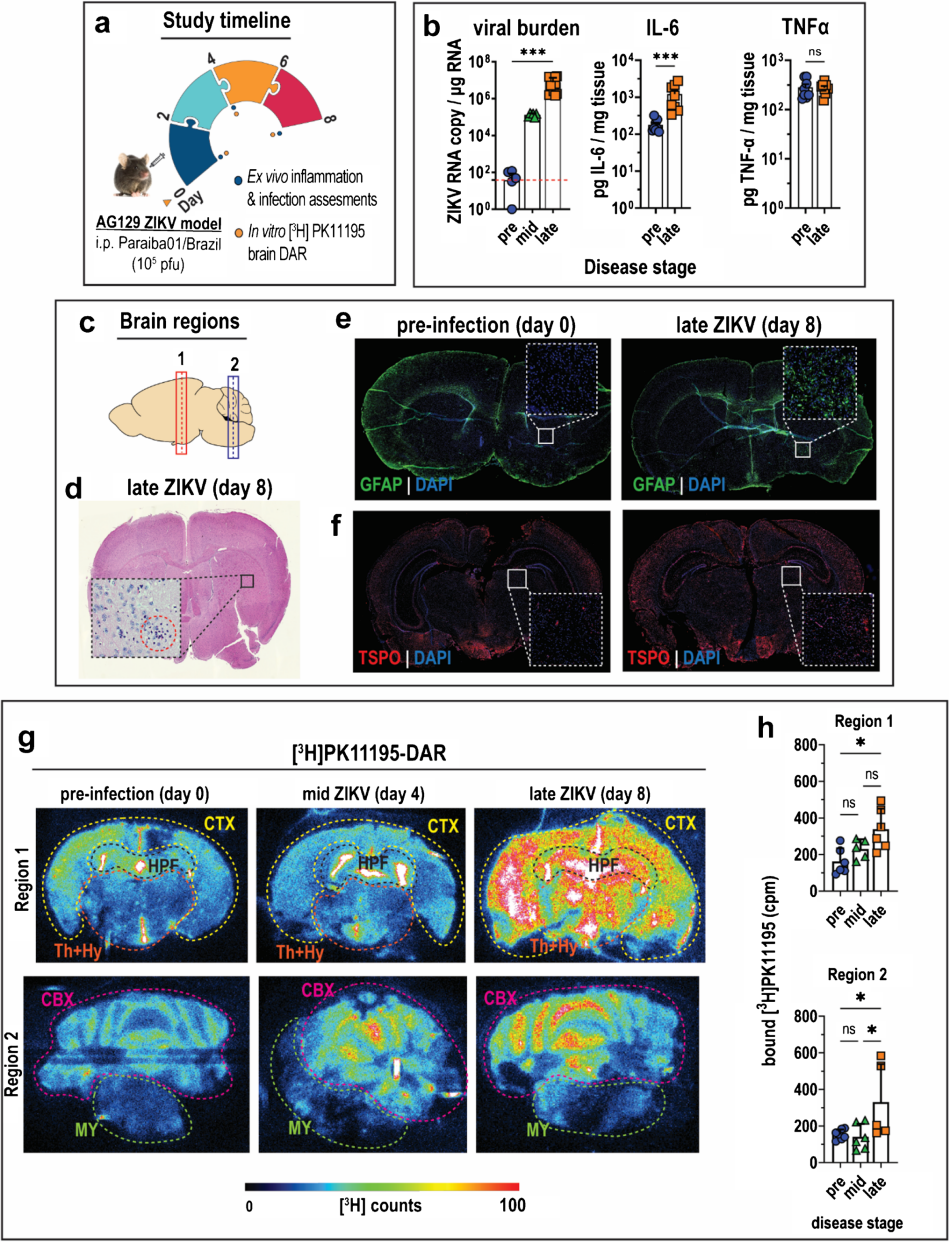
In vitro detection of neuroinflammation in ZIKV-infected mice. (**a**) Study timeline and experimental procedures performed on the acute Zika virus (ZIKV) mouse infection model. (**b–f**) Assays to detect neuroinflammation on day 0 (pre-infection), day 4 (mid disease), and day 8 (late disease). (**b**) Viral load and expression of pro-inflammatory cytokines (IL-6 and TNF-α) in whole brains harvested at stages of increasing severity of ZIKV disease. Samples were obtained from *n* = 8 mice at each disease stage. Data are presented as mean ± SD, and each point represents one mouse. Mean viral titres were compared by Kruskal–Wallis test with Dunn’s post-hoc correction; and cytokine expression was compared by Mann–Whitney test. (**c**) Brain map depicting regions of transverse slices shown in **d–g**. (**d**) Representative image of histopathologic details of neuroinflammation in late disease brains stained with hematoxylin and eosin (H&E). The encircled area highlights infiltration of immune cells in the brain parenchyma. (**e–f**) Representative images of *region 1* brain sections subjected to immunofluorescence (I.F.) staining for neuroinflammation markers (**e**) glial fibrillary acidic protein (GFAP) and (**f**) translocator protein (TSPO). The areas enclosed in white squares are enlarged in the insets. (**g**) Representative [^3^H]PK11195-DAR (digital autoradiography) images of transverse sections from brain *regions 1* and *2* taken at various stages of increasing ZIKV disease severity. Sections obtained from *n* = 6 mice at each disease stage were incubated with 0.01 mM tracer for 1 h prior to imaging. (**h**) Quantification of radioligand bound to brain tissue sections. Data are presented as mean ± SD, and individual points represent a tissue section. Means were compared by Kruskal–Wallis test with Dunn’s post-hoc correction. *p* values are displayed accordingly: *p < 0.05, ***p < 0.001. *ns*, not significant

The adult AG129 murine model of acute Zika virus (ZIKV) infection exhibited median survival of 9 days with mortality from day 8 post-infection onwards (Fig. S1a). Disease was accompanied by overt decline of health status (Fig. S1b), drastic weight loss (Fig. S1c), and neurological deficits—such as ataxia, unsteady gait, and weakness or paralysis of the limbs (Fig. S1d)—that manifested from day 4 post-infection. The study schedule was optimized to capture disease- and host response-related biomarker dynamics at increasing stages of disease severity (Fig. 1a). Increasing ZIKV burden was detected in mouse brains at mid disease (day 4) and late disease (day 8) (Fig. 1b). Disease was accompanied by hallmarks of neuroinflammation: elevated expression of pro-inflammatory cytokine interleukin-6 (IL-6) in homogenized brain tissue (Fig. 1b); infiltration of immune cells in representative tissue sections from different brain regions (Fig. 1c, d); and high expression of glial fibrillary acidic protein (GFAP) detected by immunofluorescence imaging of brain tissue sections (Fig. 1e). Moreover, increased brain expression of translocator protein (TSPO) was observed in tissue sections (Fig. 1f). Thus, ZIKV neurological infection in our model is characterized by global elevated expression of both GFAP and TSPO throughout the brain cortex and parenchyma at late ZIKV disease, which was not overt at mid disease (data not shown). These establish that ZIKV-associated neuroinflammation is accompanied by brain TSPO overexpression at late disease.

### In vitro detection of ZIKV-associated neuroinflammation with TSPO radioligand [^3^H]PK11195

Tissue sections from two selected regions (Fig. 1c) incubated with [^3^H]PK11195 and imaged by DAR (Fig. 1g) revealed twofold higher radioligand binding in late ZIKV brains compared to similarly treated pre-infection mouse brains (region 1, *p* = 0.008; region 2, *p* = 0.049) (Fig. 1h). Tracer binding was mostly localized to the cerebral cortex (CTX), hippocampal formation (HPF), diencephalon (thalamus and hypothalamus; Th + Hy), and cerebellum (CBX) (Fig. 1g). No increase in tracer binding to brain tissue was observed at mid ZIKV disease relative to pre-infection (Fig. 1h), consistent with TSPO immunostaining findings (Fig. 1f). Hence, subsequent studies focused on comparing late versus pre-disease stages.

### Ex vivo detection of ZIKV-associated neuroinflammation with TSPO radioligand [^18^F]FEPPA

**Fig. 2:**
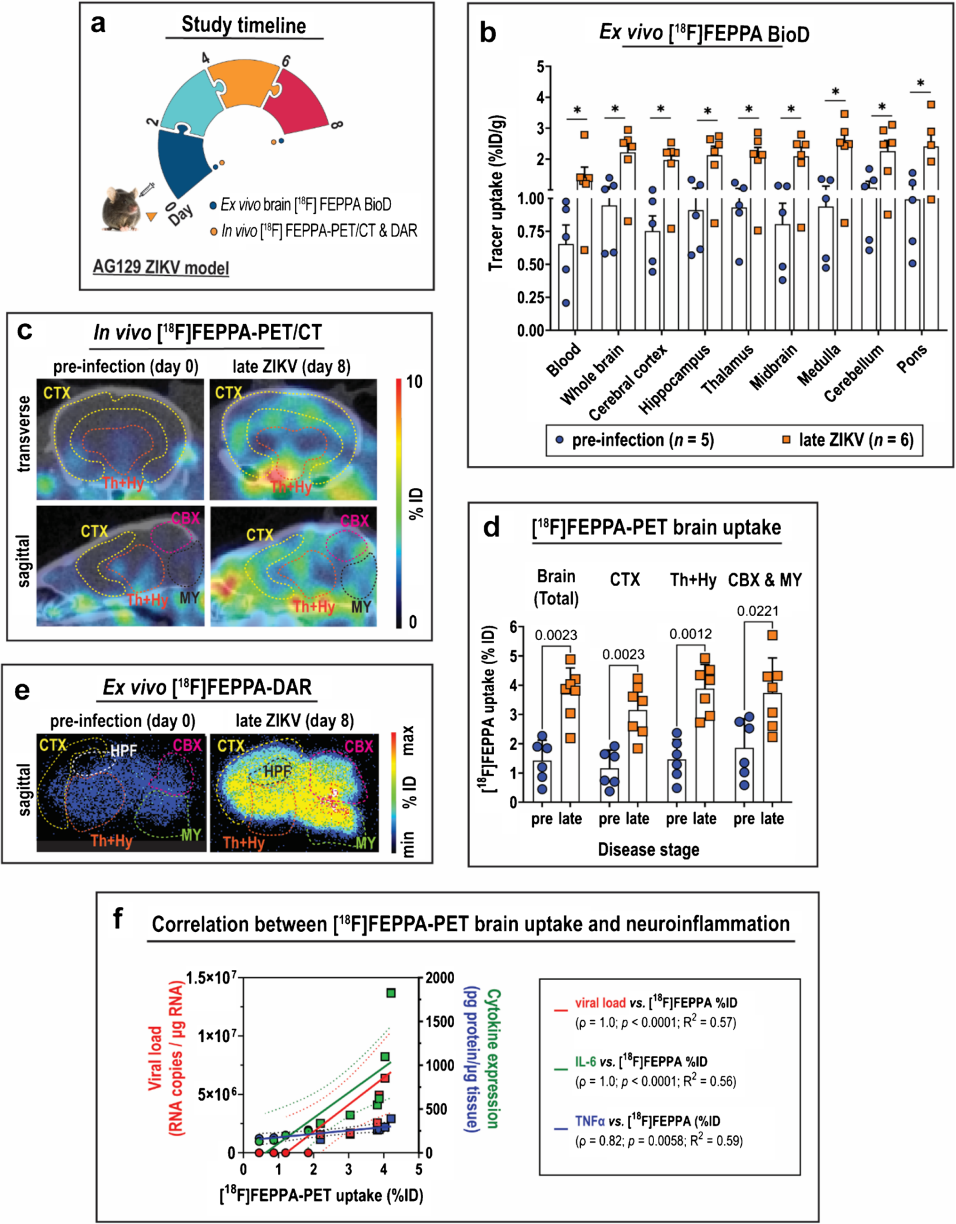
Ex vivo and in vivo detection of ZIKV-associated neuroinflammation with [^18^F]FEPPA*.* (**a**) Study timeline and experimental procedures performed on the acute Zika virus (ZIKV) mouse infection model. (**b**) Biodistribution of [^18^F]FEPPA in blood and various brain regions harvested at 2-h post-injection of tracer. Tissues were obtained from pre-infected (*n* = 5) and late ZIKV (*n* = 6) mice. Data are presented as mean ± SD, and each point represents data from individual mice. Means were compared by Mann–Whitney test, and *p* values are displayed accordingly. *p < 0.05. (**c**) Pre-infection and late disease head-focused [^18^F]FEPPA-PET/CT image slices in one animal. Representative images were taken at 2-h post-injection of tracer. Regions of interest are drawn depicting brain segmentation analysis. (**d**) Tracer uptake quantification in either whole brains or segmented brain regions reported as %ID (injected dose) in PET images. Mean %ID were compared by Mann–Whitney test. (**e**) Representative ex vivo [^18^F]FEPPA-DAR (digital autoradiography) images of sagittal brain sections harvested and sectioned at 2-h post-injection of tracer. (**f**) Correlation between brain [^18^F]FEPPA uptake and either tissue viral load or expression of pro-inflammatory cytokines IL-6 and TNF-α. Data points from pre-infection are shown as circles, and those from late disease are shown in squares. Analysis for Spearman correlation (*ρ*) was performed on scatter plots with given best-fit linear regression model (*R*^2^). The 95% confidence interval of the best-fitted regression lines are shown in correspondingly coloured dashed lines. *CTX*, cerebral cortex; *HPF*, hippocampal formation; *Th* + *Hy*, thalamus and hypothalamus (diencephalon); *CBX*, cerebellum; *MY*, medulla oblongata

Tissue biodistribution of [^18^F]FEPPA in ZIKV-infected brains was determined by ex vivo gamma counting at various stages of disease (Fig. 2a). Tracer uptake in whole brains was 2.4-fold higher at late disease versus pre-infection (2.26 vs*.* 0.95 %ID/g; *p* = 0.03) (Fig. 2b; Table S1). All micro-dissected brain regions assessed also exhibited ≥ 2.2-fold-higher [^18^F]FEPPA uptake relative to pre-infection (Fig. 2b; Table S1). However, [^18^F]FEPPA blood pool activity was notably 2.3-fold greater than pre-infection levels (1.48 vs. 0.65 %ID/g; *p* = 0.03); and brain tracer uptake at late disease was only 1.53-fold higher than in the blood (Fig. 2b; Table S1). These indicate a significant contribution of blood pool activity to whole brain or regional [^18^F]FEPPA brain uptake.

### In vivo imaging of neuroinflammation with [^18^F]FEPPA-PET/CT

[^18^F]FEPPA-PET images of ZIKV-infected brains revealed a visible increase in signal at late disease relative to pre-infection (Fig. 2c), which upon VOI analysis was 2.6-fold higher in late disease than pre-infection (3.72 vs. 1.42 %ID; *p* = 0.002) (Fig. 2d). This trend was recapitulated in the different brain regions evaluated with brain segmentation analysis: 2.7-fold increased uptake in cerebral cortex (CTX) (3.15 vs*.* 1.16 %ID; *p* = 0.002); 2.7-fold increase in the diencephalon (Th + Hy) (3.89 vs. 1.46 %ID; *p* = 0.001); and a two-fold increase in the hindbrain (CBX & MY) (3.74 vs*.* 1.86 %ID; *p* = 0.02) (Fig. 2d). The VOI volumes in PET tracer uptake analysis were comparable between groups (Fig. S2). DAR imaging after PET/CT also revealed global increased brain [^18^F]FEPPA uptake at late disease (Fig. 2e). In addition, radioligand uptake in whole brains exhibited high positive correlation with ZIKV viral load (*ρ* = 1.0; *p* < 0.0001) and expression of pro-inflammatory cytokines IL-6 (*ρ* = 1.0; *p* < 0.0001) and TNF-α (*ρ* = 0.82; *p* = 0.006) in whole brains (Fig. 2f). These strongly suggest that TSPO expression can potentially be used as a surrogate biomarker for interrogating brain tissue viral burden and neuroinflammation status in our ZIKV model by in vivo [^18^F]FEPPA-PET imaging. However, radioligand activity in the brain may also be confounded by substantial blood pool activity.

### Identification of cellular drivers of TSPO expression in ZIKV brains

**Fig. 3:**
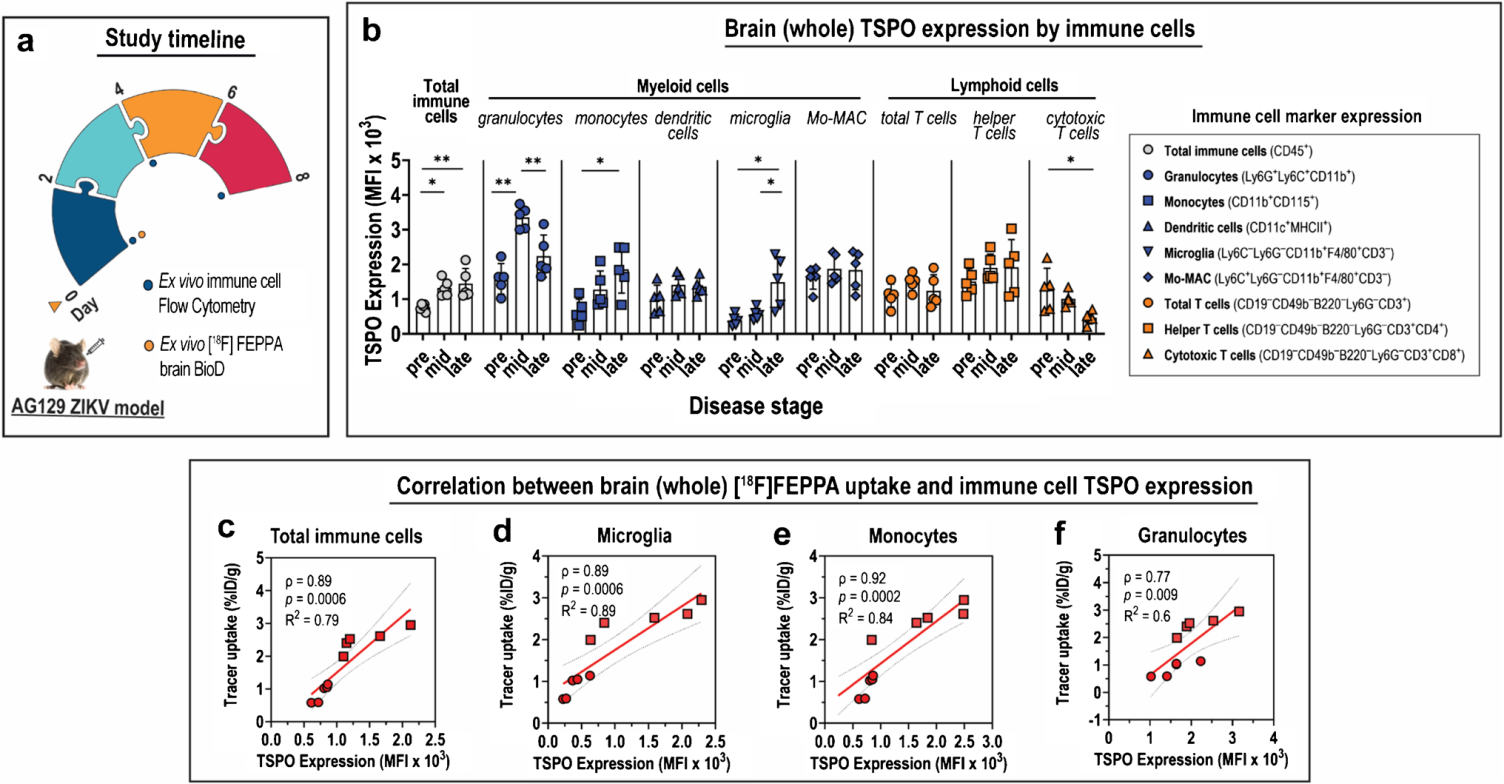
Contribution of TSPO expression on immune cells to [^18^F]FEPPA uptake in whole brains during ZIKV disease. (**a**) Study timeline and experimental procedures performed on the acute Zika virus (ZIKV) mouse infection model. (**b**) Translocator protein (TSPO) expression profile of various immune cell subsets in whole brains at various stages of Zika virus (ZIKV) disease. Immune cells were identified from *n* = 6 mice at each disease stage by flow cytometry using fluorophore-tagged antibodies for specific immune cell markers, which are shown in the legend. Data are presented as mean ± SD, and individual points represent data from individual mice. Means were compared by Kruskal–Wallis test with Dunn’s post-hoc correction. *p* values are displayed accordingly. *p < 0.05, **p < 0.005. (**c–f**) Correlation between TSPO expression of immune cells in the brain and ex vivo [^18^F]FEPPA uptake in whole brains determined by gamma counting. TSPO expression of either (**c**) total CD45^+^ immune cells, (**d**) microglia, (**e**) monocytes, and (**f**) granulocytes isolated from whole brains was determined by flow cytometry. Data points from pre-infection are shown as circles, and those from late disease are shown in squares. Analysis for Spearman correlation (*ρ*) was performed on scatter plots with given best-fit linear regression model (*R*^2^). The 95% confidence interval of the best-fitted regression lines is shown in dashed lines

TSPO-expressing cells in whole brain tissues were profiled using flow cytometry and compared with ex vivo [^18^F]FEPPA brain biodistribution analysis (Fig. 3a). Cells that do not express the general immune cell marker CD45—i.e*.*, CD45^−^ cells—exhibited low TSPO expression at < 500 mean fluorescence intensity (MFI) (Fig. S3a). These non-immune cells include neurons, neuronal support cells, and endothelia. TSPO expression on these cells did not change with increasing disease severity (Fig. S3a), and this trend was also observed in the different micro-dissected brain regions (Fig. S3b). These data signify minimal contribution of CD45^−^ cells to brain TSPO expression.

In contrast, TSPO expression in the total population of immune cells (i.e*.*, CD45^+^ cells) in the brain increased during ZIKV disease progression. Compared to pre-infection, CD45^+^ cell TSPO expression at mid and late ZIKV disease were 1.7-fold (*p* = 0.03) and 1.9-fold (*p* = 0.02) higher, respectively (Fig. 3b; Table S2a). This trend is reproduced in various micro-dissected brain regions (Fig. S4), where TSPO expression by immune cells at late disease increased by 1.3- to 3.0-fold compared to pre-infection, and the highest increase was recorded in the hippocampus and cerebral cortex (Table S3). TSPO expression in total CD45^+^ immune cells at pre and late ZIKV disease also exhibited strong correlation with ex vivo whole brain uptake of [^18^F]FEPPA (*ρ* = 0.89; *p* = 0.0006) (Fig. 3c).

The total CD45^+^ immune cells were further divided into subsets of (1) myeloid lineage and (2) lymphoid lineage cells. Myeloid lineage cells include granulocytes, monocytes, dendritic cells, brain-resident macrophages (i.e*.*, microglia), and monocyte-derived macrophages (Mo-MAC). Lymphoid lineage cells include total T cells, helper (CD4^+^) T cells, and cytotoxic (CD8^+^) T cells. Only the myeloid subset of immune cells—specifically granulocytes, monocytes, and microglia, exhibited higher TSPO expression in ZIKV disease relative to pre-infection (Fig. 3b; Table S2). TSPO expression in microglia from whole brains was 3.9-fold higher than pre-infection (*p* = 0.006) (Fig. 3b; Table S2). In various brain regions, primarily in the hippocampus and cerebellum, microglia TSPO expression increased between 2.8- and 5.7-fold (Fig. S4; Table S3). Microglia TSPO expression in whole brains exhibited strong positive correlation with [^18^F]FEPPA uptake (*ρ* = 0.89; *p* = 0.0006) (Fig. 3d). Aside from microglia, monocytes also exhibited elevated TSPO expression at late disease, which was 2.9-fold higher than pre-infection (*p* = 0.02) (Fig. 3b; Table S2). In micro-dissected brains, primarily hippocampus and cerebral cortex, the monocyte TSPO expression at late disease increased by 2.1- to 4.5-fold (Fig. S4; Table S3), with monocyte TSPO expression in whole brains strongly correlated with [^18^F]FEPPA uptake (*ρ* = 0.92; *p* = 0.0002) (Fig. 3e).

In contrast to microglia and monocytes, TSPO expression in granulocytes transiently increased at mid disease relative to pre-infection (2.1-fold higher, *p* = 0.004) (Fig. 3b, Table S2), and the strength of correlation between granulocyte TSPO expression during disease and brain tracer uptake (*ρ* = 0.77; *p* = 0.009) was weaker than either microglia or monocytes (*ρ* = 0.89–0.92) (Fig. 3f). [^18^F]FEPPA uptake in micro-dissected brains also correlated positively with TSPO expression on both total immune (CD45^+^) cells (Fig. S5a) and myeloid cell subsets—microglia, monocytes, and granulocytes (Fig. S5b) isolated from the different brain regions, replicating our observations in whole brains. These findings confirmed that TSPO expression in ZIKV brains, especially during late disease, is contributed by CD45^+^ myeloid cells—primarily monocytes and microglia.

**Fig. 4:**
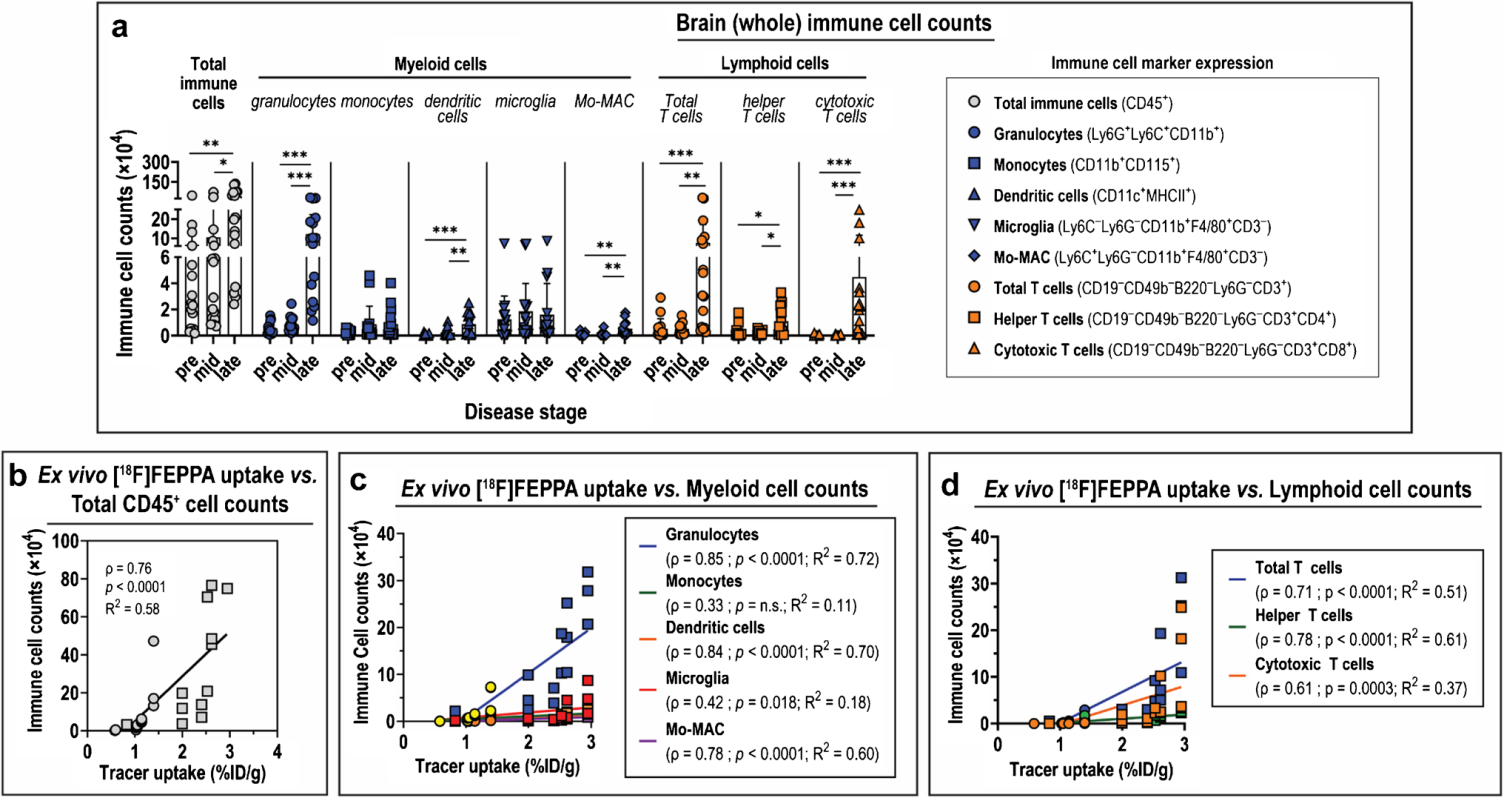
Contribution of immune cell landscape to [^18^F]FEPPA uptake in whole brains during ZIKV disease. (**a**) Absolute counts of various immune cell subsets isolated from whole brains during progressive stages of Zika virus (ZIKV) disease. Immune cells were identified from *n* = 16 mice at each disease stage by flow cytometry using fluorophore-tagged antibodies for specific immune cell markers, which are shown in the legend. Data are presented as mean ± SD, and individual points represent data from individual mice. Means were compared by Kruskal–Wallis test with Dunn’s post-hoc correction. *p* values are displayed accordingly. *p < 0.05, **p < 0.005, ***p < 0.001. (**b–d**) Correlation between ex vivo [^18^F]FEPPA uptake in whole brains and immune cell counts during ZIKV disease. (**b**) Total immune (CD45^+^) cells, (**c**) myeloid cells, and (**d**) lymphoid cells were identified by flow cytometry. Data points from pre-infection are shown as circles, and those from late disease are shown in squares. Analysis for Spearman correlation (*ρ*) was performed on scatter plots with given best-fit linear regression model (*R*.^2^)

Since TSPO is expressed by all immune cells assayed in the brain (Fig. 3b), albeit at varying levels, we hypothesized that an increase in immune cell numbers in the brain—either by infiltration from the periphery or in situ proliferation—may also contribute to the overall increase in TSPO expression in the brain. We therefore evaluated absolute counts of immune cells at different disease stages. The total CD45^+^ immune cell population in late ZIKV whole brains was 6.2-fold higher than at pre-infection (*p* < 0.001) (Fig. 4a; Table S2b). Similarly, total CD45^+^ immune cell counts at late disease increased by 3.0- to 7.2-fold in various brain regions, most prominently in the hippocampus (Fig. S6; Table S4). The trend of increased cell numbers with disease progression was also observed in different myeloid and lymphoid cell subsets. In whole brains at late disease, granulocytes increased by 21.5-fold (*p* < 0.001); dendritic cells increased by 13.5-fold (*p* < 0.001); and monocyte-derived macrophages (Mo-MAC) increased by 6.4-fold (*p* < 0.001) (Fig. 4a; Table S2b). Of the various brain regions, the hippocampus recorded the greatest fold-change increase in infiltrating myeloid cells at late disease (53-fold increase in granulocytes; 47-fold increase in dendritic cells; and 20-fold increase in Mo-MAC) (Fig. S6; Table S4). Similarly, in whole brains, total T cells at late ZIKV increased by 14.6-fold (*p* < 0.001), and helper T cells increased by 4.1-fold (*p* = 0.02). Cytotoxic T cells, which were the least abundant CD45^+^ cell subtype detected pre-infection, increased by a substantial 141.8-fold in late ZIKV disease (*p* < 0.001) (Fig. 4a; Table S2b). Of the various brain regions, the hippocampus again exhibited the highest increase in lymphoid cell counts (20-fold increase in total T cells; tenfold increase in helper T cells; and 53-fold increase in cytotoxic T cells) (Fig. S6; Table S4). Importantly, absolute counts of total CD45^+^ cells in the brain exhibited strong positive correlation with brain [^18^F]FEPPA uptake (*ρ* = 0.76; *p* < 0.0001) (Fig. 4b). Strong positive correlation was also observed between brain tracer uptake and absolute cell counts of all myeloid and lymphoid cell subsets evaluated, the most notable of which were the granulocytes, dendritic cells, and monocyte-derived macrophages (Mo-MAC) (Fig. 4c–d). These results established that increased TSPO expression in the brain is also contributed by increased numbers of immune cells—mainly the granulocytes and dendritic cells. Altogether, the TSPO expression profile and immune cell landscape in ZIKV brains provide strong support of TSPO as a biologically relevant imaging target for ZIKV neuroinflammation in our infection model.

### Determining the effect of blood cell TSPO expression on radioligand binding

**Fig. 5:**
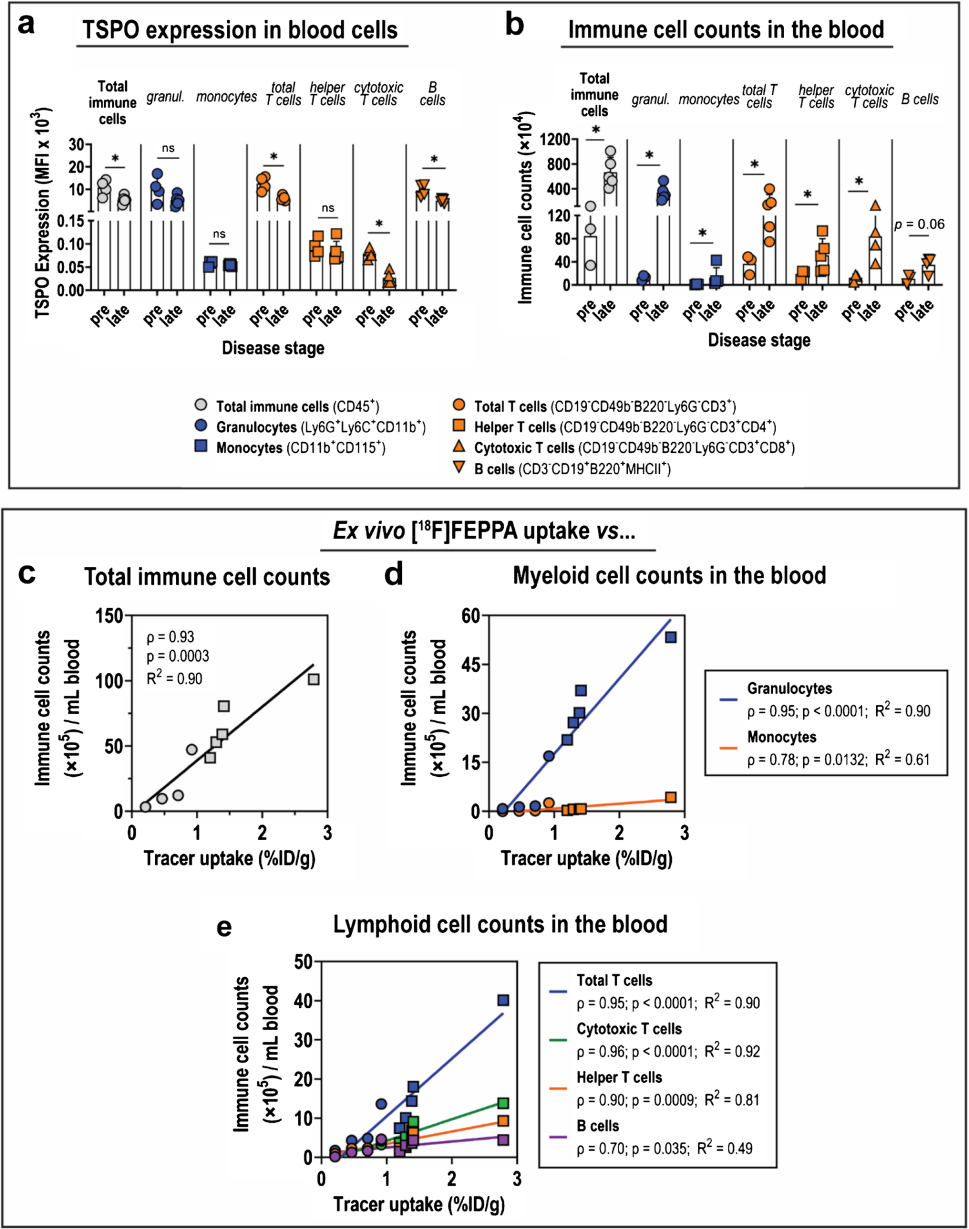
Contribution of TSPO expression on immune cells and immune cell landscape on [^18^F]FEPPA activity in the blood. (**a**) Translocator protein (TSPO) expression of various immune cells, and (**b**) Absolute counts of immune cells in the blood at various stages of Zika virus (ZIKV) disease. Immune cells were identified from *n* = 4 mice at each disease stage by flow cytometry using fluorophore-tagged antibodies for specific immune cell markers, which are shown in the legend. Data are presented as mean ± SD, and individual points represent data from individual mice. Means were compared by Mann–Whitney test. *p *values are displayed accordingly. *p < 0.05. (**c**–**e**) Correlation between ex vivo [^18^F]FEPPA activity in the blood and absolute counts of immune cells. (**c**) Total immune CD45^+^ cells, (**d**) myeloid cells, and (**e**) lymphoid cell subsets were identified by flow cytometry. Analysis for Spearman correlation (*ρ*) was performed on scatter plots with given best-fit linear regression model (*R*^2^)

Despite the demonstrated utility of both [^3^H]PK11195 and [^18^F]FEPPA to identify ZIKV-driven neuroinflammation with in vitro, ex vivo, and in vivo assays (Figs. 1–2), we noted a 2.3-fold increase in [^18^F]FEPPA blood pool activity at late disease (Fig. 2b). We therefore assessed TSPO expression in blood cells over the course of ZIKV disease. TSPO expression on total CD45^+^ immune cells in the blood decreased during ZIKV disease, and similar trends were observed with circulating granulocytes and monocytes in the blood (Fig. 5a; Table S5a). Moreover, TSPO expression in various immune cell subsets (i.e*.*, total CD45^+^ cells, myeloid, and lymphoid cells) did not correlate with blood [^18^F]FEPPA activity (Fig. S7). These contrasted with TSPO expression of these immune cells in the brain (Fig. 3b, c, f). On the other hand, total immune cell counts in the blood exhibited 3.7-fold increase in late ZIKV relative to pre-infection (*p* = 0.04), and the increased numbers were contributed primarily by granulocytes (6.6-fold increase, *p* = 0.02) and cytotoxic T cells (4.5-fold increase; *p* = 0.04) (Fig. 5b; Table S5b). There was a strong positive correlation between total CD45^+^ cell counts in the blood and [^18^F]FEPPA blood uptake (*ρ* = 0.93; *p* = 0.0003) (Fig. 5c), and this trend was reflected in granulocytes (*ρ* = 0.95; *p* < 0.0001), monocytes (*ρ* = 0.78; *p* = 0.01) (Fig. 5d), as well as lymphoid cells evaluated (Fig. 5e). These findings demonstrate that the increased blood pool [^18^F]FEPPA activity in late ZIKV disease mice is due to the increased presence of true TSPO targets.

## Discussion

Zika virus (ZIKV) disease is associated with acute neuroinflammation caused by viral insults to the central nervous system (CNS). ZIKV brain infection is accompanied by hallmarks of neuroinflammation, such as the elevated protein expression of glial fibrillary acidic protein (GFAP) and translocator protein (TSPO) in the CNS [20, 38]. We observed in this AG129 model of acute ZIKV disease a pattern of globally elevated brain TSPO expression relative to healthy brains by TSPO-immunostaining and [^3^H]PK11195-DAR on tissue sections. Ex vivo tissue distribution of [^18^F]FEPPA revealed 2.4-fold higher binding to late disease brains compared to pre-infection controls, which corroborated with 2.6-fold signal enhancement in [^18^F]FEPPA-PET/CT at late disease. PET signal enhancement was also observed in the cerebral cortex, midbrain, diencephalon, and hindbrain by brain segmentation image analysis. Thus, brain TSPO expression correlates with ZIKV neuroinflammation, and [^18^F]FEPPA brain uptake can be used as a surrogate marker for neuroinflammation in ZIKV disease. TSPO-PET could be potentially useful in identifying patients with CNS involvement from a pool of ZIKV-infected asymptomatic or mild individuals. Additionally, this imaging biomarker can expedite development of ZIKV disease-ameliorating therapeutics by identifying which drugs can cross the blood–brain barrier, exhibit potent antiviral activity, and ameliorate neuroinflammation.

We also identified subsets of cells driving increased TSPO expression in ZIKV-infected brains. Aside from microglia—which are brain-resident immune cells—astrocytes, dopaminergic neurons, and vascular endothelia have been accepted as sources of brain TSPO expression in acute and chronic neuroinflammation (reviewed in [22, 39]). The contribution of other immune cells to brain TSPO expression, especially in the context of viral encephalitis, has been grossly understudied. ZIKV is an acute inflammatory disease accompanied by a massive influx of immune cells into the affected tissues [24, 27, 40]. Our study established that CD45^+^ immune cells, and not CD45^−^ cells, primarily express TSPO in ZIKV brains. At late disease, microglia and CNS-infiltrating monocytes exhibited 3.9-fold and 2.9-fold increased TSPO expression, respectively. TSPO expression in monocytes is worth noting since human monocytes are highly susceptible to ZIKV infection [41].

Flow cytometry further revealed infiltration of immune cells into the brain, especially the hippocampus. The nearly 50-fold enhancement of dendritic cells in the hippocampus is impressive given its small size relative to other brain regions (e.g*.*, cerebral cortex). However, this is not surprising since the adult hippocampus contains the highest density of neuroprogenitor cells targeted by ZIKV infection [24, 42, 43]. In addition, dendritic cells were shown as primary targets of ZIKV infection in the blood which are then used as Trojan horses to transport virus into the brain [44, 45]. In whole brains and dissected brain regions, both myeloid and lymphoid immune cell subsets were increased in late ZIKV disease. Indeed, the immune landscape changes in ZIKV-infected AG129 mouse brains are consistent with previous observations of increased inflammatory macrophages, neutrophils, and T cells in both wild-type and interferon-deficient model (Ifnar1^−/−^) of ZIKV-infected mice [40, 46].

ZIKV disease in the AG129 model is characterized by high demand for immune cells in the brain and spleen [27], which leads to increased presence of immune cells in circulation. We observed > fourfold increase in the number of TSPO-expressing immune cells in the blood at late disease. This supports the twofold increased ex vivo [^18^F]FEPPA blood activity at late disease relative to pre-infection, and indicates that immune cells represent a true TSPO target for radioligand binding in the blood. Thus, in vivo [^18^F]FEPPA-PET imaging of ZIKV-associated neuroinflammation in our model is due to specific binding to TSPO targets in blood immune cells with some contribution for non-specific [^18^F]FEPPA binding to serum proteins [47]. Therefore, brain TSPO-PET in ZIKV infection and other viral encephalitis models will need to be accompanied by appropriate image analysis methods, such as cardiac input correction [47], to separate blood pool activity from specific PET signals originating from the brain. Moreover, due to the complexity of disease, in particular the diversity of TSPO-expressing cells involved in response to ZIKV infection, future studies need to be adequately statistically powered to observe tracer uptake difference between groups. Study sample size will be a crucial consideration in detecting treatment response by [^18^F]FEPPA-PET imaging.

Our study is the first to fully validate TSPO expression as a biologically relevant imaging target for ZIKV neuroinflammatory disease and to demonstrate that elevated brain TSPO expression in late ZIKV disease is conferred by two mechanisms: (1) increased TSPO expression on myeloid cells, primarily infiltrating monocytes and brain-resident microglia; and (2) overwhelming increase in numbers of TSPO-expressing immune cells, mainly of granulocytes and T cells. [^18^F]FEPPA could distinguish ZIKV neuroinflammation in an interferon receptor-deficient AG129 mouse model, which exhibits increased brain TSPO expression that correlates with disease severity. [^18^F]FEPPA brain uptake, which is contributed by microglia and other immune cells infiltrating the brain, is a potential surrogate non-invasive imaging biomarker for neuroinflammation in this model. Future studies on virus-induced neuroinflammation and related encephalitis must also investigate the role of immune cells.

## Supplementary Information

Below is the link to the electronic supplementary material.

Supplementary file 1 (DOCX 3.69 MB)

## Data Availability

All raw data and materials are available upon request.
