# Supplementary material for: TSPO expression in a Zika virus murine infection model as an imaging target for acute infection-induced neuroinflammation
Source: Eur J Nucl Med Mol Imaging. 2022 Nov 9;50(3):742–55. doi: 10.1007/s00259-022-06019-w (PMC9852192; doi:10.1007/s00259-022-06019-w)
Supplement: Supplementary file 1 — (DOCX 3.69 MB) [file 259_2022_6019_MOESM1_ESM.docx]

*Original Article (Infection and Inflammation)*

**TSPO expression in a Zika virus murine infection model as an imaging target for acute infection-induced neuroinflammation**

Carla Bianca Luena Victorio^1,^*, Rasha Msallam^1^, Wisna Novera^1^, Joanne Ong^1^, Tham Jing Yang^1^,

Arun Ganasarajah^1^, Jenny Low^2,3^, Satoru Watanabe^3^, Ann-Marie Chacko^1,^*

Laboratory for Translational and Molecular Imaging, Cancer and Stem Cell Biology Programme, Duke-NUS Medical School, Singapore

^2^Department of Infectious Diseases, Singapore General Hospital, Singapore

^3^Programme in Emerging Infectious Disease, Duke-NUS Medical School, Singapore

*Correspondence to:

Ann-Marie Chacko, PhD, Assistant Professor, Cancer & Stem Cell Biology Programme; Head, Laboratory for Translational and Molecular Imaging (LTMI), Duke-NUS Medical School, 8 College Road, Singapore, 167952. Email: [ann-marie.chacko@duke-nus.edu.sg](mailto:ann-marie.chacko@duke-nus.edu.sg)

Carla Bianca Luena Victorio, PhD, Senior Research Fellow, Cancer & Stem Cell Biology Programme, Duke-NUS Medical School, 8 College Road, Singapore, 167952. Email: [carla-bianca.victorio@duke-nus.edu.sg](mailto:carla-bianca.victorio@duke-nus.edu.sg)

For submission to: European Journal of Nuclear Medicine and Molecular Imaging

*Original Article (Infection and Inflammation)*

Keywords: Zika, neuroinflammation, translocator protein, TSPO, PET

**Supplemental Files**

**Table S1.** *Brain tissue distribution of [^18^F]FEPPA in late ZIKV disease* v.s. *pre-infection.* Mice were injected with 2 MBq [^18^F]FEPPA at either pre-infection (day 0) or late Zika virus (ZIKV) disease (day 8 post-infection), and euthanized at 120 min post-tracer injection. Tissue biodistribution data are summarized and presented in **Fig. 2b**. Tracer uptake between groups was compared by Mann-Whitney test. *n.s.*, not significant.

|  | Tracer uptake % ID/g ± SD | | | |
| --- | --- | --- | --- | --- |
| Tissue | **Pre**  **disease** | **Late**  **disease** | **Fold-change**  **late *vs.* pre** | ***p*-value** |
| Blood | 0.65 ± 0.32 | 1.48 ± 0.80 | 2.3 | 0.03 |
| Whole brain | 0.95 ± 0.36 | 2.26 ± 0.83 | 2.4 | 0.03 |
| Cerebral cortex (CTX) | 0.75 ± 0.26 | 1.99 ± 0.7 | 2.6 | 0.03 |
| Hippocampus (HPF) | 0.91 ± 0.34 | 2.18 ± 0.79 | 2.4 | 0.03 |
| Thalamus | 0.93 ± 0.27 | 2.10 ± 0.81 | 2.3 | 0.05 |
| Midbrain | 0.80 ± 0.36 | 2.13 ± 0.79 | 2.7 | 0.03 |
| Medulla | 0.94 ± 0.42 | 2.42 ± 0.99 | 2.6 | 0.03 |
| Cerebellum | 1.08 ± 0.45 | 2.38 ± 0.88 | 2.2 | 0.03 |
| Pons | 0.99 ± 0.42 | 2.41 ± 1.51 | 2.4 | 0.03 |

**Table S2.** *Translocator protein (TSPO) expression and immune cell landscape in the brains during ZIKV disease.* Whole mouse brains were harvested at pre (day 0), mid (day 4) and late (day 8) Zika virus (ZIKV) disease and processed for flow cytometry as described in ***Materials and Methods*** section. (**a**) TSPO expression and (**b**) absolute immune cell counts were determined and normalized to pre disease. Myeloid cells are highlighted in blue, while lymphoid cells are highlighted in orange. Data for TSPO expression are summarized and presented in **Fig. 3b**; and for immune cell counts in **Fig. 4a**. Means were compared by Kruskal-Wallis test with Dunn’s post-hoc correction. *n.s.*, not significant; *MFI*, mean fluorescence intensity.

| 1. TSPO expression Mean Fluorescence Intensity (MFI) × 10^3^ | | | | | | | |
| --- | --- | --- | --- | --- | --- | --- | --- |
| *Immune cells* | ***pre*** | ***mid disease (day 4)*** | | | ***late disease (day 8)*** | | |
|  | **mean± SD** | **mean± SD** | **Fold-change *vs*. pre** | ***p*-value** | **mean± SD** | **Fold-change *vs*. pre** | ***p*-value** |
| Total CD45^+^ | 0.77 ± 0.11 | 1.33 ± 0.24 | 1.7 | 0.03 | 1.45 ± 0.44 | 1.9 | 0.02 |
| Granulocytes | 1.59 ± 0.44 | 3.36 ± 0.32 | 2.1 | 0.004 | 2.24 ± 0.61 | 1.4 | *n.s.* |
| Monocytes | 0.65 ± 0.35 | 1.28 ± 0.54 | 2.0 | *n.s.* | 1.86 ± 0.69 | 2.9 | 0.02 |
| Dendritic cells | 1.0 ± 0.40 | 1.42 ± 0.31 | 1.4 | *n.s.* | 1.36 ± 0.25 | 1.4 | *n.s.* |
| Microglia | 0.39 ± 0.16 | 0.57 ± 0.17 | 1.5 | *n.s.* | 1.49 ± 0.73 | 3.9 | 0.006 |
| Mo-MAC | 1.62 ± 0.33 | 1.87 ± 0.42 | 1.2 | *n.s.* | 1.84 ± 0.56 | 1.1 | *n.s.* |
| Total T cells | 1.10 ± 0.33 | 1.49 ± 0.24 | 1.3 | *n.s.* | 1.24 ± 0.47 | 1.1 | *n.s.* |
| Helper T cells | 1.51 ± 0.37 | 1.90 ± 0.40 | 1.3 | *n.s.* | 1.92 ± 0.80 | 1.3 | *n.s.* |
| Cytotoxic T cells | 1.27 ± 0.62 | 1.00 ± 0.21 | 0.8 | *n.s.* | 0.47 ± 0.18 | 0.37 | 0.02 |

| 1. Immune cell counts × 10^4^ | | | | | | | |
| --- | --- | --- | --- | --- | --- | --- | --- |
| *Immune cells* | ***pre*** | ***mid disease (day 4)*** | | | ***late disease (day 8)*** | | |
|  | **mean± SD** | **mean± SD** | **Fold-change *vs*. pre** | ***p*-value** | **mean± SD** | **Fold-change *vs*. pre** | ***p*-value** |
| Total CD45^+^ | 6.70 ± 11.82 | 10.57 ± 18.95 | 1.6 | *n.s.* | 41.8 ± 44.59 | 6.2 | *<0.001* |
| Granulocytes | 0.57 ± 0.48 | 0.85 ± 0.57 | 1.5 | *n.s.* | 12.26 ± 10.1 | 21.5 | *<0.001* |
| Monocytes | 0.26 ± 0.21 | 0.87 ± 1.38 | 3.3 | *n.s.* | 0.89 ± 1.12 | 3.7 | *<0.001* |
| Dendritic cells | 0.07 ± 0.08 | 0.23 ± 0.30 | 3.5 | *n.s.* | 0.89 ± 0.81 | 13.5 | *<0.001* |
| Microglia | 1.26 ± 1.77 | 1.86 ± 2.13 | 1.86 | *n.s.* | 1.61 ± 2.38 | 1.3 | *n.s.* |
| Mo-MAC | 0.08 ± 0.13 | 0.10 ± 0.18 | 1.2 | *n.s.* | 0.54 ± 0.53 | 6.4 | *<0.001* |
| Total T cells | 0.53 ± 0.77 | 0.52 ± 0.46 | 1.0 | *n.s.* | 7.71 ± 9.54 | 14.6 | *<0.001* |
| Helper T cells | 0.27 ± 0.47 | 0.17 ± 0.18 | 0.7 | *n.s.* | 1.92 ± 0.80 | 1.3 | *n.s.* |
| Cytotoxic T cells | 0.03 ± 0.06 | 0.03 ± 0.04 | 0.9 | *n.s.* | 0.47 ± 0.18 | 0.37 | 0.02 |

**Table S3.** *Translocator protein (TSPO) expression contributed by immune cells in various brain regions during ZIKV disease.* Brains were collected from late (day 8) and mid (day 4) Zika virus (ZIKV) disease and micro-dissected into cerebral cortex (CTX), cerebellar cortex (CBX), hippocampal formation (HPF), midbrain (MB), diencephalon (Dien), and pons and medulla (P+M). Isolated cells were processed for flow cytometry as described in ***Materials and Methods*** section. TSPO expression was determined and normalized to pre disease. Data are summarized and presented in **Fig. S4-S5.** Mean TSPO expression between groups was compared by Mann-Whitney test. *n.s.*, not significant.

|  |  | TSPO expression Mean Fluorescence Intensity (MFI) × 10^3^ | | | | | | |
| --- | --- | --- | --- | --- | --- | --- | --- | --- |
| *Immune cells* | ***Brain region*** | ***pre*** | ***mid disease (day 4)*** | | | ***late disease (day 8)*** | | |
|  |  | **mean± SD** | **mean± SD** | **Fold-change *vs.* pre** | ***p*-value** | **mean± SD** | **Fold-change *vs.* pre** | ***p*-value** |
| Total CD45^+^ | **CTX** | 0.61 ± 0.53 | 1.68 ± 1.85 | 2.8 | *n.s.* | 1.20 ± 1.09 | 2.0 | *n.s.* |
| Total CD45^+^ | **CBX** | 0.85 ± 0.34 | 1.28 ± 0.85 | 1.5 | *n.s.* | 1.66 ± 0.68 | 1.9 | *n.s.* |
| Total CD45^+^ | **HPF** | 0.72 ± 0.32 | 1.10 ± 0.85 | 1.5 | *n.s.* | 2.13 ± 0.58 | 3.0 | 0.002 |
| Total CD45^+^ | **MB+Dien** | 0.81 ± 0.54 | 1.12 ± 0.41 | 1.4 | *n.s.* | 1.15 ± 0.88 | 1.4 | *n.s.* |
| Total CD45^+^ | **P+M** | 0.86 ± 0.49 | 1.45 ± 1.06 | 1.7 | *n.s.* | 1.10 ± 0.79 | 1.3 | *n.s.* |
|  |  |  |  |  |  |  |  |  |
| Granulocytes | **CTX** | 1.02 ± 0.77 | 2.59 ± 2.38 | 2.5 | *n.s.* | 1.65 ± 1.48 | 1.6 | *n.s.* |
| Granulocytes | **CBX** | 1.64 ± 0.44 | 3.55 ± 0.72 | 2.2 | 0.002 | 2.53 ± 0.75 | 1.6 | *n.s.* |
| Granulocytes | **HPF** | 2.23 ± 0.25 | 3.74 ± 1.13 | 1.7 | 0.03 | 3.16 ± 1.26 | 1.4 | *n.s.* |
| Granulocytes | **MB+Dien** | 1.41 ± 0.30 | 3.00 ± 0.82 | 2.1 | 0.03 | 1.96 ± 1.73 | 1.4 | *n.s.* |
| Granulocytes | **P+M** | 1.64 ± 0.12 | 3.44 ± 0.14 | 2.1 | 0.009 | 1.89 ± 0.51 | 1.2 | *n.s.* |
|  |  |  |  |  |  |  |  |  |
| Monocytes | **CTX** | 0.24 ± 0.21 | 0.85 ± 0.81 | 3.6 | *n.s.* | 0.84 ± 0.62 | 3.5 | 0.02 |
| Monocytes | **CBX** | 1.17 ± 0.81 | 1.54 ± 0.43 | 1.3 | *n.s.* | 2.49 ± 0.56 | 2.1 | 0.007 |
| Monocytes | **HPF** | 0.55 ± 0.06 | 0.90 ± 0.30 | 1.6 | *n.s.* | 2.48 ± 1.01 | 4.5 | <0.001 |
| Monocytes | **MB+Dien** | 0.51 ± 0.14 | 1.00 ± 0.41 | 2.0 | *n.s.* | 1.64 ± 1.37 | 3.2 | 0.04 |
| Monocytes | **P+M** | 0.78 ± 0.53 | 2.10 ± 0.23 | 2.7 | 0.01 | 1.84 ± 0.78 | 2.4 | *n.s.* |
|  |  |  |  |  |  |  |  |  |
| Dendritic cells | **CTX** | 0.59 ± 0.41 | 1.17 ± 0.86 | 2.0 | *n.s.* | 1.07 ± 0.94 | 1.8 | *n.s.* |
| Dendritic cells | **CBX** | 1.06 ± 0.73 | 1.33 ± 0.84 | 1.3 | *n.s.* | 1.42 ± 0.15 | 1.3 | *n.s.* |
| Dendritic cells | **HPF** | 1.59 ± 0.98 | 1.69 ± 0.82 | 1.0 | *n.s.* | 1.73 ± 0.58 | 1.1 | *n.s.* |
| Dendritic cells | **MB+Dien** | 1.10 ± 0.39 | 1.79 ± 0.83 | 1.6 | *n.s.* | 1.31 ± 0.93 | 1.2 | *n.s.* |
| Dendritic cells | **P+M** | 0.66 ± 0.32 | 1.09 ± 0.75 | 1.7 | *n.s.* | 1.25 ± 0.49 | 1.9 | *n.s.* |
|  |  |  |  |  |  |  |  |  |
| Microglia | **CTX** | 0.23 ± 0.26 | 0.40 ± 0.38 | 1.7 | *n.s.* | 0.64 ± 0.42 | 2.8 | 0.006 |
| Microglia | **CBX** | 0.63 ± 0.53 | 0.83 ± 0.33 | 1.3 | *n.s.* | 2.29 ± 0.67 | 3.7 | 0.002 |
| Microglia | **HPF** | 0.37 ± 0.08 | 0.55 ± 0.28 | 1.5 | *n.s.* | 2.08 ± 0.78 | 5.7 | <0.001 |
| Microglia | **MB+Dien** | 0.44 ± 0.21 | 0.60 ± 0.18 | 1.4 | *n.s.* | 1.59 ± 1.05 | 3.6 | 0.01 |
| Microglia | **P+M** | 0.27 ± 0.28 | 0.46 ± 0.42 | 1.7 | *n.s.* | 0.84 ± 0.41 | 3.1 | *n.s.* |
|  |  |  |  |  |  |  |  |  |
| Mo-MAC | **CTX** | 1.88 ± 0.57 | 1.60 ± 1.00 | 0.9 | *n.s.* | 1.09 ± 0.64 | 0.6 | *n.s.* |
| Mo-MAC | **CBX** | 1.66 ± 0.89 | 2.36 ± 0.79 | 1.4 | *n.s.* | 2.03 ± 0.64 | 1.2 | *n.s.* |
| Mo-MAC | **HPF** | 1.84 ± 0.74 | 1.46 ± 1.11 | 0.8 | *n.s.* | 2.37 ± 0.84 | 1.3 | *n.s.* |
| Mo-MAC | **MB+Dien** | 1.64 ± 0.61 | 2.28 ± 1.56 | 1.4 | *n.s.* | 2.29 ± 1.41 | 1.4 | *n.s.* |
| Mo-MAC | **P+M** | 1.06 ± 0.73 | 1.66 ± 1.23 | 1.6 | *n.s.* | 1.40 ± 0.40 | 1.3 | *n.s.* |
|  |  |  |  |  |  |  |  |  |
| Total T cells | **CTX** | 0.64 ± 0.57 | 1.54 ± 1.67 | 2.4 | *n.s.* | 1.01 ± 0.87 | 1.6 | *n.s.* |
| Total T cells | **CBX** | 1.17 ± 0.58 | 1.79 ± 0.64 | 1.5 | *n.s.* | 1.53 ± 0.18 | 1.3 | *n.s.* |
| Total T cells | **HPF** | 1.56 ± 1.23 | 1.12 ± 0.96 | 0.7 | *n.s.* | 1.90 ± 0.26 | 1.2 | *n.s.* |
| Total T cells | **MB+Dien** | 1.13 ± 0.70 | 1.43 ± 0.50 | 1.3 | *n.s.* | 0.96 ± 0.79 | 0.9 | *n.s.* |
| Total T cells | **P+M** | 1.02 ± 0.60 | 1.57 ± 1.17 | 1.5 | *n.s.* | 0.77 ± 0.64 | 0.8 | *n.s.* |
|  |  |  |  |  |  |  |  |  |
| Helper T cells | **CTX** | 1.28 ± 1.43 | 1.60 ± 1.82 | 1.3 | *n.s.* | 1.20 ± 0.84 | 0.93 | *n.s.* |
| Helper T cells | **CBX** | 2.03 ± 1.11 | 2.13 ± 0.58 | 1.1 | *n.s.* | 2.23 ± 0.95 | 1.1 | *n.s.* |
| Helper T cells | **HPF** | 1.45 ± 1.64 | 2.50 ± 1.52 | 1.7 | *n.s.* | 3.03 ± 1.53 | 2.1 | *n.s.* |
| Helper T cells | **MB+Dien** | 1.70 ± 1.55 | 1.67 ± 0.76 | 1.0 | *n.s.* | 2.04 ± 1.58 | 1.2 | *n.s.* |
| Helper T cells | **P+M** | 1.09 ± 0.68 | 1.62 ± 1.32 | 1.5 | *n.s.* | 1.08 ± 0.80 | 1.0 | *n.s.* |
|  |  |  |  |  |  |  |  |  |
| Cytotoxic T cells | **CTX** | 0.66 ± 0.56 | 0.91 ± 0.88 | 1.4 | *n.s.* | 0.44 ± 0.50 | 0.66 | *n.s.* |
| Cytotoxic T cells | **CBX** | 1.41 ± 0.47 | 1.34 ± 0.56 | 1.0 | *n.s.* | 0.47 ± 0.29 | 0.34 | *n.s.* |
| Cytotoxic T cells | **HPF** | 2.19 ± 0.94 | 1.03 ± 0.61 | 0.5 | *n.s.* | 0.70 ± 0.36 | 0.30 | <0.001 |
| Cytotoxic T cells | **MB+Dien** | 1.36 ± 0.51 | 0.98 ± 0.44 | 0.7 | *n.s.* | 0.52 ± 0.56 | 0.40 | 0.02 |
| Cytotoxic T cells | **P+M** | 0.72 ± 0.66 | 0.77 ± 0.74 | 1.1 | *n.s.* | 0.20 ± 0.14 | 0.30 | *n.s.* |

**Table S4.** *Immune cell landscape in various brain regions during ZIKV disease.* Brains were collected from late (day 8) and mid (day 4) Zika virus (ZIKV) disease and micro-dissected into cerebral cortex (CTX), cerebellar cortex (CBX), hippocampal formation (HPF), midbrain (MB), diencephalon (Dien), and pons and medulla (P+M). Isolated cells were processed for flow cytometry as described in ***Materials and Methods*** section. Immune cell counts were determined and normalized to pre disease. Data are summarized and presented in **Fig. S4-S5.** Mean cell counts between groups were compared by Mann-Whitney test. *n.s.*, not significant.

|  |  | Absolute cell counts ×10^4^ | | | | | | |
| --- | --- | --- | --- | --- | --- | --- | --- | --- |
| *Immune cells* | ***Brain region*** | ***pre*** | ***mid disease (day 4)*** | | | ***late disease (day 8)*** | | |
|  |  | **mean± SD** | **mean± SD** | **Fold-change *vs.* pre** | ***p*-value** | **mean± SD** | **Fold-change *vs.* pre** | ***p*-value** |
| Total CD45 | **CTX** | 1.14 ± 2.08 | 1.88 ± 2.83 | 1.7 | *n.s.* | 6.19 ± 7.06 | 5.4 | 0.008 |
| Total CD45 | **CBX** | 1.03 ± 1.94 | 2.70 ± 8.67 | 2.6 | *n.s.* | 3.41 ± 8.77 | 3.3 | *n.s.* |
| Total CD45 | **HPF** | 0.40 ± 0.61 | 0.35 ± 0.44 | 0.9 | *n.s.* | 2.61 ± 2.76 | 6.6 | 0.001 |
| Total CD45 | **MB+Dien** | 3.05 ± 5.97 | 4.30 ± 8.20 | 1.4 | *n.s.* | 17.21 ± 21.14 | 5.6 | 0.008 |
| Total CD45 | **P+M** | 1.16 ± 2.45 | 1.34 ± 1.53 | 1.2 | *n.s.* | 8.38 ± 9.01 | 7.2 | <0.001 |
|  |  |  |  |  |  |  |  |  |
| Granulocytes | **CTX** | 0.07 ± 0.11 | 0.17 ± 0.16 | 2.6 | *n.s.* | 2.37 ± 2.73 | 36.2 | <0.001 |
| Granulocytes | **CBX** | 0.04 ± 0.07 | 0.08 ± 0.13 | 1.9 | *n.s.* | 0.40 ± 0.55 | 9.0 | 0.002 |
| Granulocytes | **HPF** | 0.01 ± 0.02 | 0.06 ± 0.11 | 4.2 | <0.001 | 0.73 ± 0.94 | 53.3 | <0.001 |
| Granulocytes | **MB+Dien** | 0.32 ± 0.47 | 0.34 ± 0.25 | 1.0 | *n.s.* | 5.88 ± 5.62 | 18.4 | <0.001 |
| Granulocytes | **P+M** | 0.07 ± 0.08 | 0.12 ± 0.12 | 1.7 | *n.s.* | 2.93 ± 4.04 | 43.9 | <0.001 |
|  |  |  |  |  |  |  |  |  |
| Monocytes | **CTX** | 0.25 ± 0.63 | 0.28 ± 0.46 | 1.1 | *n.s.* | 0.25 ± 0.43 | 1.0 | *n.s.* |
| Monocytes | **CBX** | 0.04 ± 0.06 | 0.03 ± 0.04 | 0.8 | *n.s.* | 0.06 ± 0.12 | 1.5 | *n.s.* |
| Monocytes | **HPF** | 0.07 ± 0.12 | 0.05 ± 0.07 | 0.7 | *n.s.* | 0.12 ± 0.22 | 1.9 | *n.s.* |
| Monocytes | **MB+Dien** | 0.34 ± 0.77 | 0.44 ± 0.98 | 1.3 | *n.s.* | 0.19 ± 0.31 | 0.6 | *n.s.* |
| Monocytes | **P+M** | 0.12 ± 0.31 | 0.07 ± 0.07 | 0.6 | *n.s.* | 0.27 ± 0.62 | 2.2 | *n.s.* |
|  |  |  |  |  |  |  |  |  |
| Dendritic cells | **CTX** | 0.01 ± 0.02 | 0.02 ± 0.03 | 1.7 | *n.s.* | 0.33 ± 0.42 | 27.8 | <0.001 |
| Dendritic cells | **CBX** | 0.01 ± 0.01 | 0.01 ± 0.01 | 0.83 | *n.s.* | 0.05 ± 0.05 | 6.1 | 0.01 |
| Dendritic cells | **HPF** | 0.00 ± 0.00 | 0.00 ± 0.01 | 1.5 | *n.s.* | 0.11 ± 0.11 | 47.2 | <0.001 |
| Dendritic cells | **MB+Dien** | 0.04 ± 0.07 | 0.20 ± 0.31 | 4.9 | 0.02 | 0.14 ± 0.28 | 3.5 | *n.s.* |
| Dendritic cells | **P+M** | 0.01 ± 0.01 | 0.01 ± 0.01 | 0.8 | *n.s.* | 0.17 ± 0.23 | 20.3 | <0.001 |
|  |  |  |  |  |  |  |  |  |
| Microglia | **CTX** | 0.43 ± 0.88 | 0.83 ± 1.6 | 1.9 | *n.s.* | 0.53 ± 1.04 | 1.2 | *n.s.* |
| Microglia | **CBX** | 0.12 ± 0.17 | 0.11 ± 0.12 | 0.9 | *n.s.* | 0.08 ± 0.09 | 0.7 | *n.s.* |
| Microglia | **HPF** | 0.17 ± 0.37 | 0.07 ± 0.12 | 0.4 | *n.s.* | 0.42 ± 0.82 | 2.5 | *n.s.* |
| Microglia | **MB+Dien** | 0.28 ± 0.30 | 0.38 ± 0.32 | 1.3 | *n.s.* | 0.45 ± 0.67 | 1.6 | *n.s.* |
| Microglia | **P+M** | 0.05 ± 0.06 | 0.26 ± 0.46 | 4.8 | *n.s.* | 0.17 ± 0.22 | 3.2 | *n.s.* |
|  |  |  |  |  |  |  |  |  |
| Mo-MAC | **CTX** | 0.01 ± 0.01 | 0.02 ± 0.02 | 2.2 | *n.s.* | 0.54 ± 0.53 | 6.4 | <0.001 |
| Mo-MAC | **CBX** | 0.00 ± 0.00 | 0.00 ± 0.01 | 1.4 | *n.s.* | 0.03 ± 0.04 | 12.8 | 0.006 |
| Mo-MAC | **HPF** | 0.00 ± 0.00 | 0.00 ± 0.00 | 0.7 | *n.s.* | 0.06 ± 0.07 | 20.3 | <0.001 |
| Mo-MAC | **MB+Dien** | 0.04 ± 0.07 | 0.04 ± 0.05 | 0.8 | *n.s.* | 0.08 ± 0.07 | 1.8 | *n.s.* |
| Mo-MAC | **P+M** | 0.00 ± 0.01 | 0.01 ± 0.01 | 1.3 | *n.s.* | 0.18 ± 0.28 | 42.5 | <0.001 |
|  |  |  |  |  |  |  |  |  |
| Total T cells | **CTX** | 0.05 ± 0.06 | 0.08 ± 0.09 | 1.7 | *n.s.* | 0.83 ± 1.14 | 17.9 | <0.001 |
| Total T cells | **CBX** | 0.05 ± 0.07 | 0.04 ± 0.05 | 0.8 | *n.s.* | 0.24 ± 0.34 | 4.5 | *n.s.* |
| Total T cells | **HPF** | 0.02 ± 0.02 | 0.04 ± 0.06 | 2.3 | *n.s.* | 0.34 ± 0.42 | 20.4 | <0.001 |
| Total T cells | **MB+Dien** | 0.17 ± 0.20 | 0.22 ± 0.28 | 1.3 | *n.s.* | 1.79 ± 1.80 | 10.3 | 0.01 |
| Total T cells | **P+M** | 0.13 ± 0.29 | 0.14 ± 0.14 | 1.1 | *n.s.* | 1.77 ± 2.13 | 13.5 | 0.001 |
|  |  |  |  |  |  |  |  |  |
| Helper T cells | **CTX** | 0.03 ± 0.04 | 0.04 ± 0.06 | 1.5 | *n.s.* | 0.14 ± 0.21 | 5.4 | *n.s.* |
| Helper T cells | **CBX** | 0.02 ± 0.02 | 0.01 ± 0.01 | 0.8 | *n.s.* | 0.06 ± 0.09 | 4.0 | *n.s.* |
| Helper T cells | **HPF** | 0.01 ± 0.01 | 0.01 ± 0.01 | 1.5 | *n.s.* | 0.05 ± 0.07 | 10.0 | 0.003 |
| Helper T cells | **MB+Dien** | 0.17 ± 0.37 | 0.08 ± 0.12 | 0.5 | *n.s.* | 0.64 ± 0.80 | 3.7 | 0.03 |
| Helper T cells | **P+M** | 0.05 ± 0.13 | 0.04 ± 0.04 | 0.7 | *n.s.* | 0.26 ± 0.27 | 4.8 | <0.001 |
|  |  |  |  |  |  |  |  |  |
| Cytotoxic T cells | **CTX** | 0.01 ± 0.01 | 0.01 ± 0.02 | 1.7 | *n.s.* | 0.17 ± 0.26 | 3.16 | 0.02 |
| Cytotoxic T cells | **CBX** | 0.00 ± 0.00 | 0.00 ± 0.00 | 1.0 | *n.s.* | 0.05 ± 0.10 | 34.8 | 0.01 |
| Cytotoxic T cells | **HPF** | 0.00 ± 0.00 | 0.00 ± 0.00 | 0.3 | *n.s.* | 0.07 ± 0.12 | 53.4 | 0.004 |
| Cytotoxic T cells | **MB+Dien** | 0.01 ± 0.02 | 0.01 ± 0.02 | 1.0 | *n.s.* | 3.07 ± 4.90 | 251.5 | <0.001 |
| Cytotoxic T cells | **P+M** | 0.01 ± 0.03 | 0.00 ± 0.01 | 0.4 | *n.s.* | 0.91 ± 1.44 | 76.0 | <0.001 |

**Table S5.** *Translocator protein (TSPO) expression and immune cell landscape in the blood during ZIKV disease.* Blood was collected on late (day 8) Zika virus (ZIKV) disease and processed for flow cytometry as described in ***Materials and Methods*** section. (**a**) TSPO expression and (**b**) absolute immune cell counts were determined and normalized to pre disease. Myeloid cells are highlighted in blue, while lymphoid cells are highlighted in orange. Data for TSPO expression are summarized and presented in **Fig. 5a**; and for immune cell counts in **Fig. 5b**. Means were compared by Mann-Whitney test. *n.s.*, not significant; *MFI*, mean fluorescence intensity.

|  | 1. TSPO expression Mean Fluorescence Intensity (MFI) × 10^3^ | | | |
| --- | --- | --- | --- | --- |
| *Immune cells* | ***pre*** | ***late disease (day 8)*** | | |
|  | **mean± SD** | **mean± SD** | **Fold-change *vs.* pre** | ***p*-value** |
| Total CD45^+^ | 10.78 ± 3.52 | 5.40 ± 1.68 (0.50; *0.03*) | 0.50 | 0.03 |
| Granulocytes | 10.18 ± 5.63 | 5.13 ± 2.32 (0.50; *n.s.*) | 0.50 | *n.s.* |
| Monocytes | 0.06 ± 0.01 | 0.05 ± 0.003 (0.94; *n.s.*) | 0.94 | *n.s.* |
| T cells | 12.59 ± 3.12 | 6.09 ± 1.04 (0.48; *0.02*) | 0.48 | 0.02 |
| Helper T cells | 0.09 ± 0.02 | 0.08 ± 0.02 (0.90; *n.s.*) | 0.90 | *n.s.* |
| Cytotoxic T cells | 0.08 ± 0.01 | 0.03 ± 0.01 (0.33; *0.02*) | 0.33 | 0.02 |
| B cells | 9.52 ± 2.76 | 4.95 ± 0.87 (14.6; *0.02*) | 14.6 | 0.02 |

|  | (b) Absolute cell counts ×10^5^ per ml blood | |  |  |
| --- | --- | --- | --- | --- |
| *Immune cells* | ***pre*** | ***late disease (day 8)*** |  |  |
|  | **mean± SD** | **mean± SD** | **Fold-change *vs.* pre** | ***p*-value** |
| Total CD45^+^ | 18.17 ± 19.78 | 66.90 ± 23.85 | 3.7 | 0.04 |
| Granulocytes | 5.15 ± 7.84 | 33.92 ± 12.15 | 6.6 | 0.04 |
| Monocytes | 0.72 ± 1.24 | 1.32 ± 1.67 | 1.8 | 0.04 |
| T cells | 6.14 ± 5.17 | 18.02 ± 13.00 | 2.9 | 0.04 |
| Helper T cells | 2.44 ± 1.35 | 5.11 ± 2.88 | 2.1 | 0.04 |
| Cytotoxic T cells | 1.74 ± 1.15 | 7.74 ± 3.95 | 4.5 | 0.04 |
| B cells | 1.95 ± 1.90 | 3.40 ± 1.20 | 1.8 | *n.s.* |

**Figure S1**

**
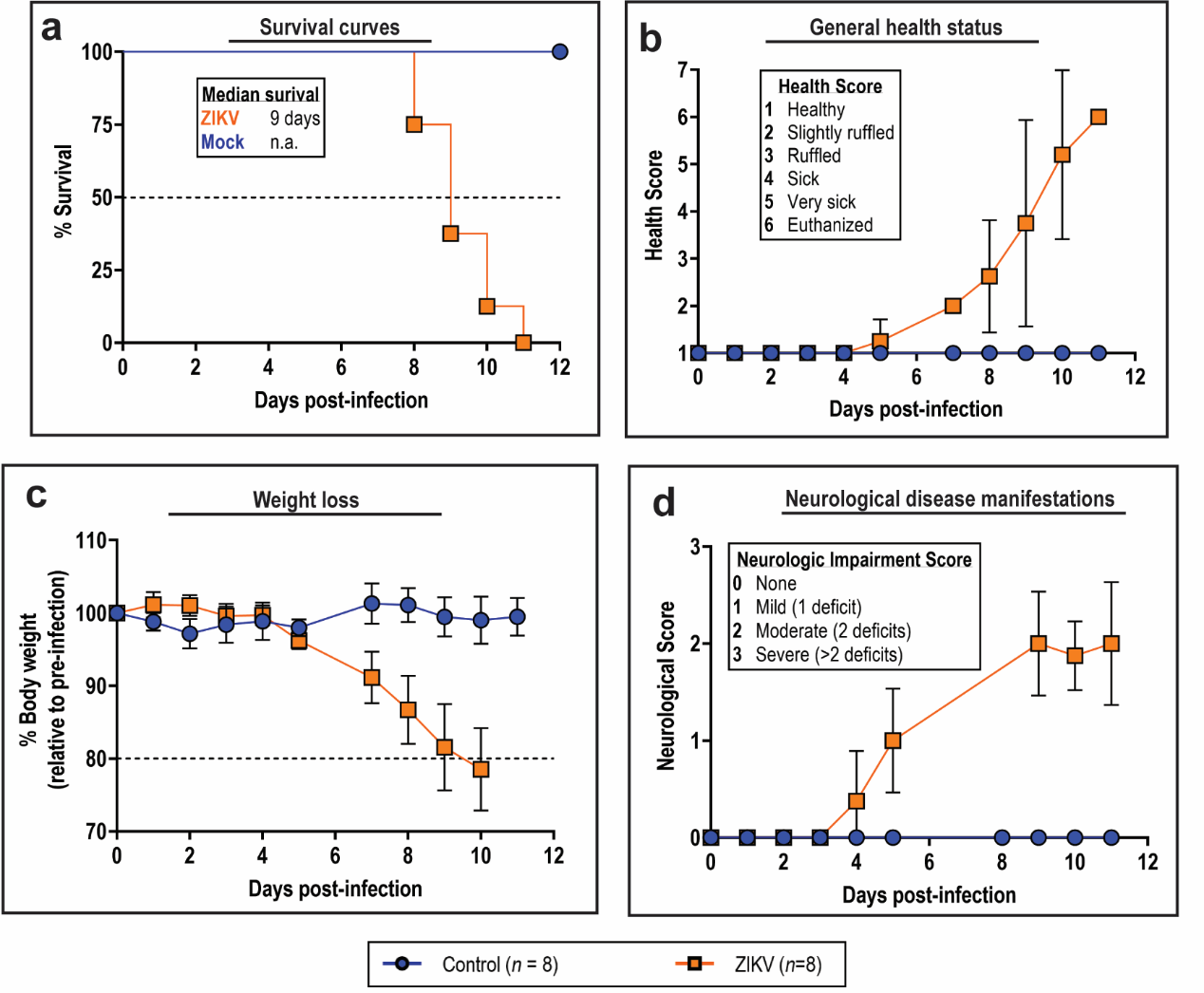
**

**Fig. S1**. *Characteristics of the murine ZIKV neuroinflammation model*. (**a**) Kaplan-Meier survival curve, (**b**) general health scores, (**c**) changes in body weight relative to pre-infection, and (**d**) neurological impairment scores in mice recorded over 12 days. Mice were either inoculated with Zika virus (ZIKV) or sham-infected (Control). The dashed lines in **a** represent the median survival, while the dashed lines in **c** represent the maximum weight loss required for euthanasia. The health scoring system was adapted from Sumathy *et. al.* 2017 [[1](#_ENREF_1)], while the scoring system for neurological impairment was adapted from Guy *et. al.*, 2007 [[2](#_ENREF_2)].

**Figure S2**

**
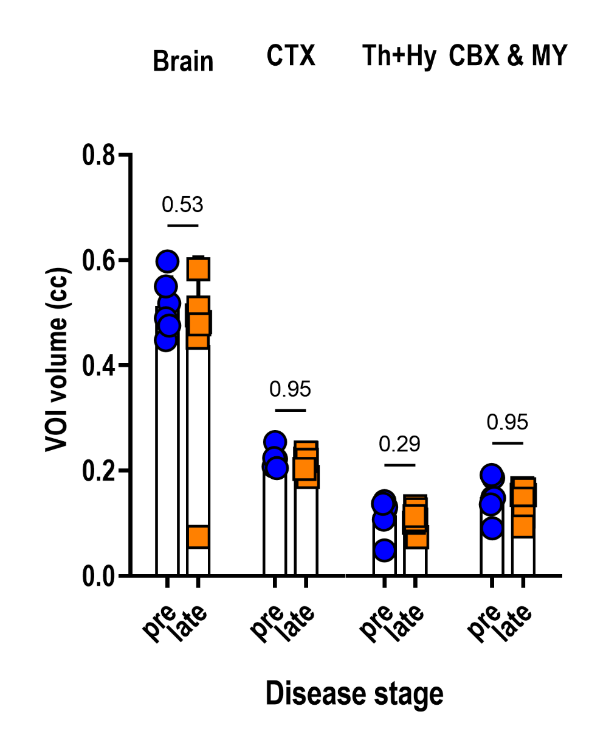
**

**Fig. S2**. *Comparison of VOI volumes used in brain segmentation analysis of [^18^F]FEPPA uptake.* Volumes of interest (VOI) were drawn around either total brain using the CT image of the skull as a guide. Similarly, VOIs around the various brain sub-regions were drawn using CT landmarks. Mean volumes were compared with Mann-Whitney test. *CTX*, cerebral cortex. *Th+Hy*, thalamus and hypothalamus (diencephalon). CBX, cerebellar cortex. MY, medulla.

**Figure S3**

**
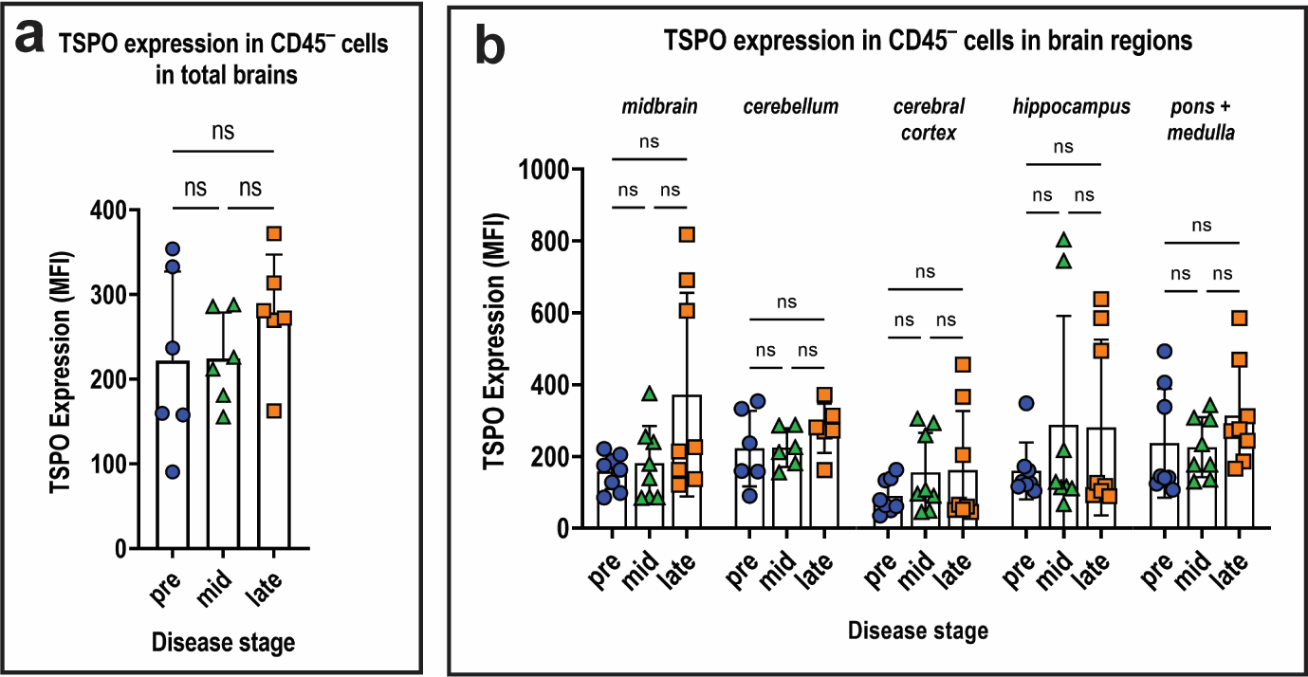
**

**Fig. S3**. *TSPO expression in CD45-negative cells within brain tissues.* TSPO expression on CD45^−^ cells from either (**a**) total brain tissues or (**b**) micro-dissected brains into various regions. TSPO expression is measured in mean fluorescence intensity (MFI) of detected signals from TSPO-specific antibodies bound on cells of interest. Data are presented as mean ± SD, and each point represents data from individual mice. Means were compared by Kruskal-Wallis test with Dunn’s post-hoc correction.. *n.s.*, not significant.

**Figure S4**

**
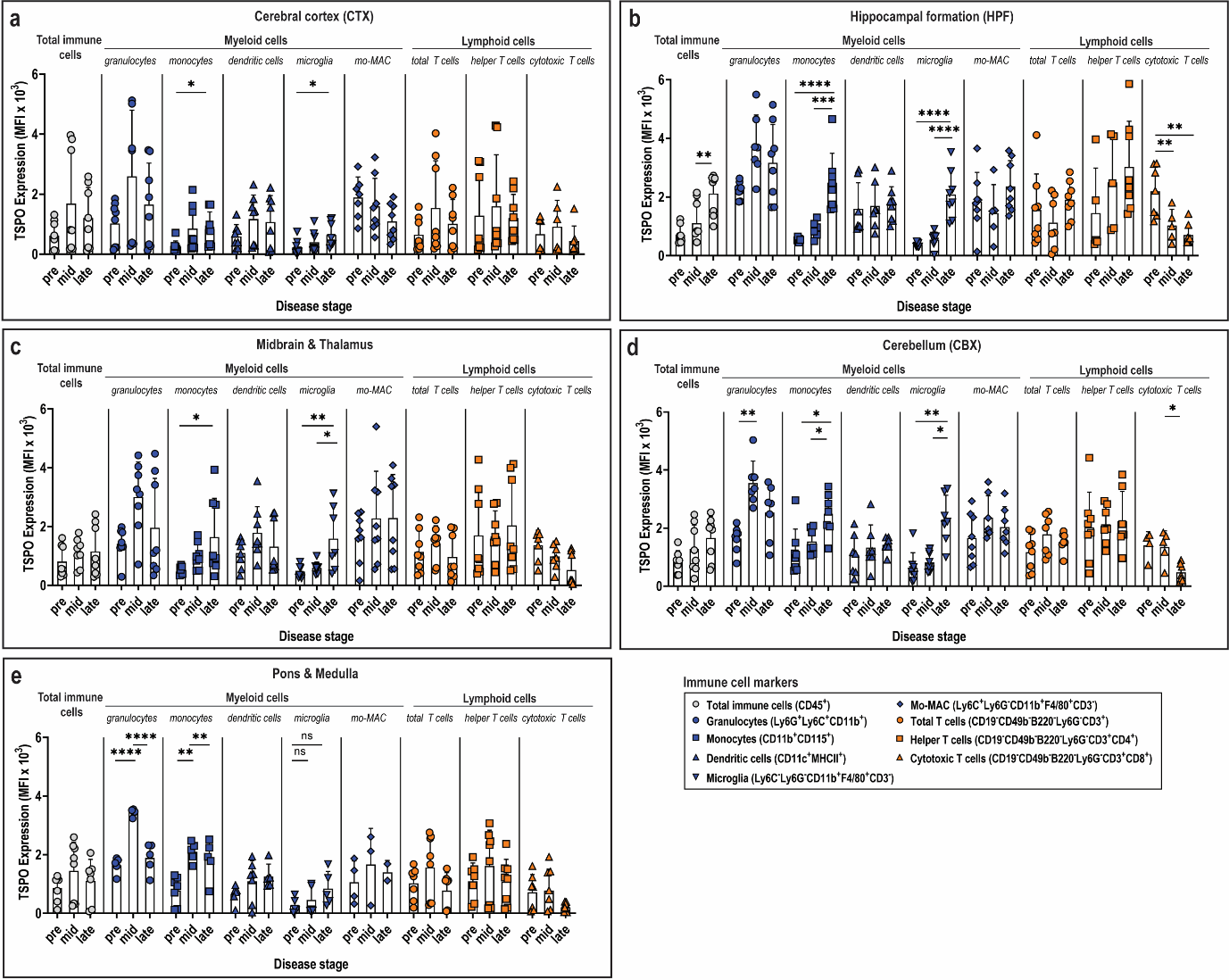
**

**Fig. S4**. *TSPO expression on immune cells isolated from various regions of mouse ZIKV* *brains.* Whole brains were harvested at pre-infection (day 0), mid (day 4), and late Zika virus (ZIKV) disease (day 8 post-infection) and micro-dissected to isolate the (**a**) cerebral cortex, (**b**) hippocampus, (**c**) midbrain and thalamus, (**d**) cerebellum, and (**e**) medulla and pons. Immune cells were identified by flow cytometry using fluorophore-tagged antibodies for specific immune cell markers, which are shown in the legend. Data are presented as mean ± SD, and individual points represent data from individual mice. TSPO expression between groups was compared by Kruskal-Wallis test with Dunn’s post-hoc correction, and *p*-values are displayed accordingly. * *p* < 0.05. ** *p* <0.005. *** *p* <0.001.

**Figure S5**

**
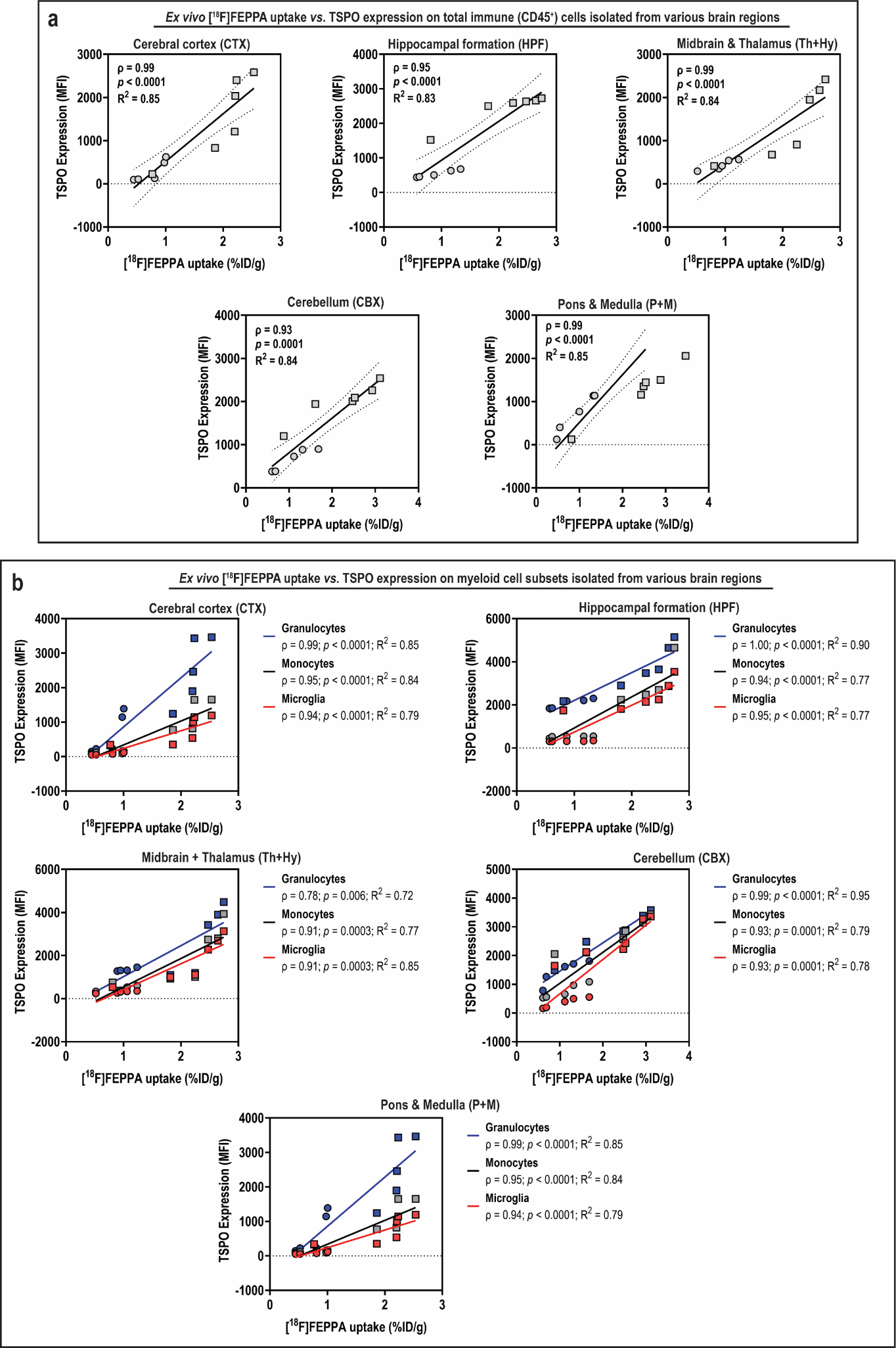
**

**Fig. S5**. *Correlation between ex vivo [^18^F]FEPPA uptake and TSPO expression on immune cells isolated from various brain regions*. Whole brains were harvested at pre-infection (day 0) and late Zika virus (ZIKV) disease (day 8) and micro-dissected into sub-regions. Immune cells were identified by flow cytometry using fluorophore-tagged antibodies for specific immune cell markers. Spearman correlation between tracer uptake and TSPO expression on (**a**) total immune (CD45^+^) cell; and (**b**) myeloid cell subpopulation was determined. Data points from pre-infection are shown as circles, and those from late disease are shown in squares. Analysis for Spearman correlation (ρ) was performed on scatter plots with given best-fit linear regression model (R^2^).

**Figure S6**

**
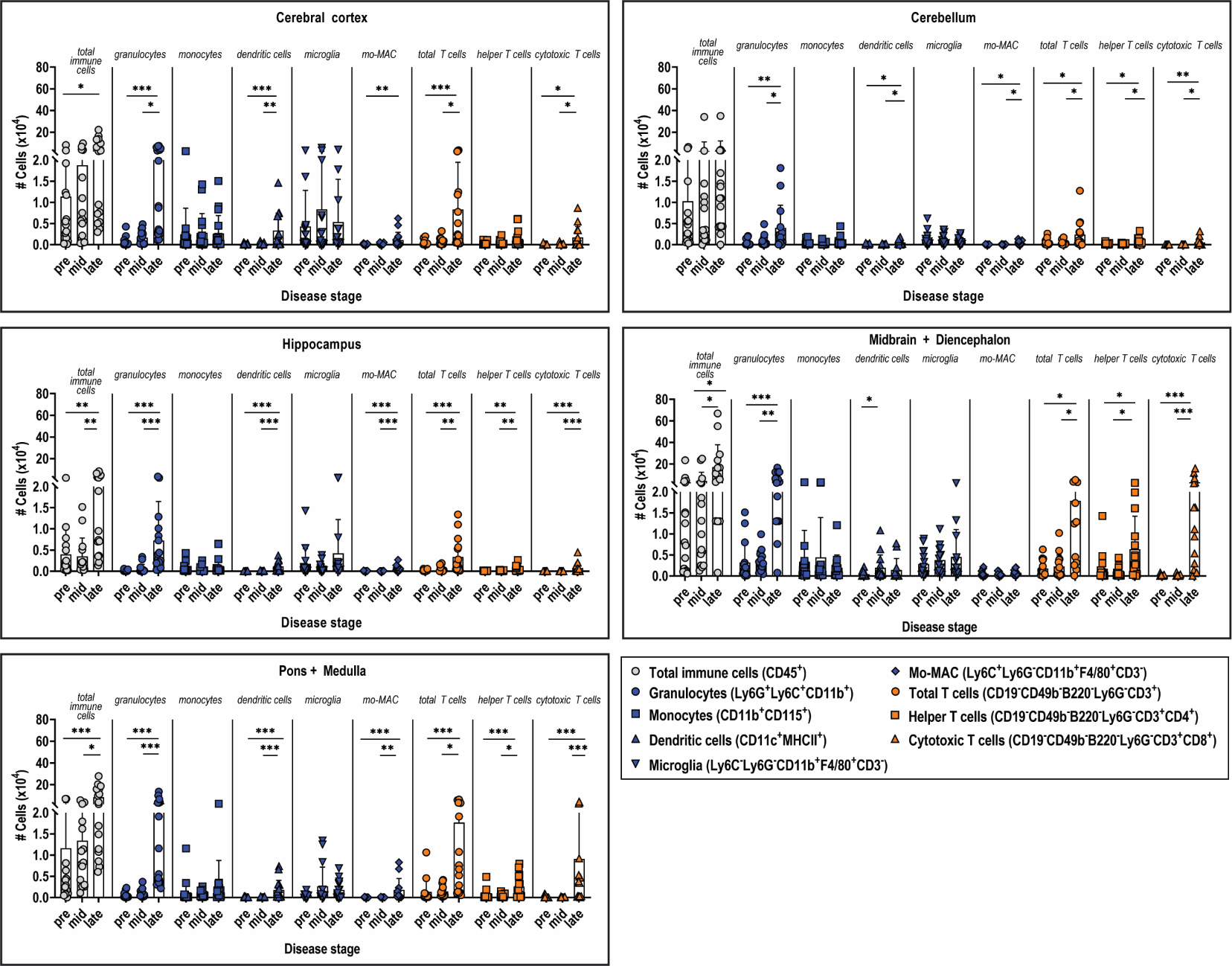
**

**Fig. S6**. *Absolute cell counts of various immune cells isolated from different regions of ZIKV mouse brains*. Whole brains were harvested at pre-infection (day 0), mid (day 4), and late Zika virus (ZIKV) disease (day 8 post-infection). Immune cells were identified by flow cytometry using fluorophore-tagged antibodies for specific immune cell markers, which are shown in the Legend. Data are presented as mean ± SD, and individual points represent data from individual mice. Means were compared Kruskal-Wallis test with Dunn’s post-hoc correction, and *p*-values are displayed accordingly. * *p* < 0.05. ** *p* <0.005. *** *p* <0.001.

**Figure S7**

**
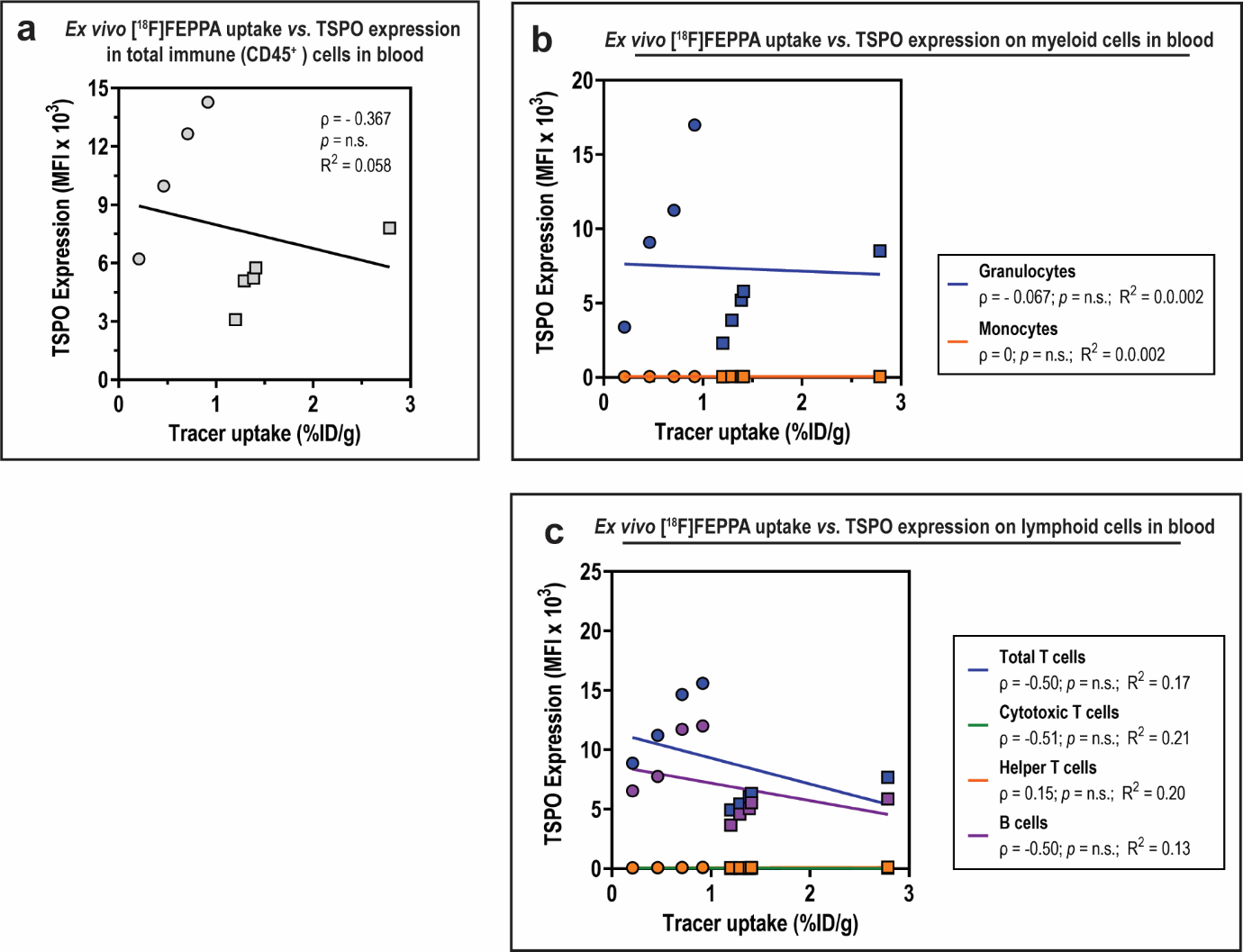
**

**Fig. S7**. *Correlation between [^18^F]FEPPA blood pool activity and TSPO expression on blood immune cells*. Spearman correlation between tracer uptake and TSPO expression on (**a**) total immune (CD45^+^) cell; (**b**) myeloid cell subpopulation; and (**c**) lymphoid cell subpopulation. Data points from pre-infection are shown as circles, and those from late disease are shown in squares. Analysis for Spearman correlation (ρ) was performed on scatter plots with given best-fit linear regression model (R^2^).

**References**

1. Sumathy K, Kulkarni B, Gondu RK, Ponnuru SK, Bonguram N, Eligeti R, et al. Protective efficacy of Zika vaccine in AG129 mouse model. Sci Rep. 2017;7:46375. doi:10.1038/srep46375.

2. Guy J, Gan J, Selfridge J, Cobb S, Bird A. Reversal of neurological defects in a mouse model of Rett syndrome. Science. 2007;315:1143-7. doi:10.1126/science.1138389.
